# Prevalence of Borderline Personality Disorder in University Samples: Systematic Review, Meta-Analysis and Meta-Regression

**DOI:** 10.1371/journal.pone.0155439

**Published:** 2016-05-12

**Authors:** Rebecca Meaney, Penelope Hasking, Andrea Reupert

**Affiliations:** 1 Faculty of Education, Monash University, Melbourne, Australia; 2 School of Psychology & Speech Pathology, Curtin University, Perth, Australia; Central Institute of Mental Health, GERMANY

## Abstract

***Objective:*** To determine pooled prevalence of clinically significant traits or features of Borderline Personality Disorder among college students, and explore the influence of methodological factors on reported prevalence figures, and temporal trends. ***Data Sources:*** Electronic databases (1994–2014: AMED; Biological Abstracts; Embase; MEDLINE; PsycARTICLES; CINAHL Plus; Current Contents Connect; EBM Reviews; Google Scholar; Ovid Medline; Proquest central; PsychINFO; PubMed; Scopus; Taylor & Francis; Web of Science (1998–2014), and hand searches. ***Study Selection*:** Forty-three college-based studies reporting estimates of clinically significant BPD symptoms were identified (5.7% of original search). ***Data Extraction:*** One author (RM) extracted clinically relevant BPD prevalence estimates, year of publication, demographic variables, and method from each publication or through correspondence with the authors. ***Results:*** The prevalence of BPD in college samples ranged from 0.5% to 32.1%, with lifetime prevalence of 9.7% (95% CI, 7.7–12.0; p < .005). Methodological factors contributing considerable between-study heterogeneity in univariate meta-analyses were participant anonymity, incentive type, research focus and participant type. Study and sample characteristics related to between study heterogeneity were sample size, and self-identifying as Asian or “other” race. The prevalence of BPD varied over time: 7.8% (95% CI 4.2–13.9) between 1994 and 2000; 6.5% (95% CI 4.0–10.5) during 2001 to 2007; and 11.6% (95% CI 8.8–15.1) from 2008 to 2014, yet was not a source of heterogeneity (*p =* .09). ***Conclusions:*** BPD prevalence estimates are influenced by the methodological or study sample factors measured. There is a need for consistency in measurement across studies to increase reliability in establishing the scope and characteristics of those with BPD engaged in tertiary study.

## Introduction

Borderline Personality Disorder (BPD) is associated with adverse and persistent psychological symptoms that are greater in severity among young people.[1] Specific to symptoms, people with the disorder may engage in self-harm, experience recurrent suicidal ideation, and in 10% of cases, die by suicide.[1] Additionally, BPD diminishes capacity for successful interpersonal relationships, results in difficulty regulating emotional states, and interrupts cognitive processes essential for learning and memory acquisition.[2] Subsequently, those who are impacted by BPD may experience difficulties in cognitive and psychosocial functioning, both of which underpin a successful college study experience.[3] It has been suggested that BPD symptoms can be reliably found in college student populations,[3] however the scope of the issue has been difficult to quantify. To date, there has not been an attempt to estimate pooled prevalence of BPD in college populations, or examine the influence of methodology on prevalence rates, and such investigation may be warranted. BPD has been associated with lower education levels, [4,5] and particular risk of attrition at university-level study.[3,4,6] As such, establishing prevalence of BPD in college students, may serve to quantify a population at risk of poor academic outcomes, and potentially justify the allocation of college-based mental health resources in response.

There have been considerable differences in estimates of clinically relevant BPD symptoms in college populations with reported figures between 0.5%[7] and 32.1%. [8] While it has been suggested that the prevalence of BPD is increasing over time,[9] it is unclear whether this represents a reliable phenomenon, or simply reflects significant variations in the methodology employed. Measurement is commonly cited as a cause for heterogeneity across studies, with structured diagnostic interviews typically yielding lower prevalence rates, and self-report measures thought to result in over-reporting of disorders.[10,11] Moreover, there is considerable variance across self-report measures of BPD, related to measurement domains, number of items, response format (scale versus dichotomous), and time period assessed. For example, the Borderline Evaluation of Severity Over Time (BEST) scale[12] allows the administrator to assess symptoms over periods as brief as seven days. As BPD is associated with emotional lability, it may be that assessment of symptoms over shorter time frames result in over or under-reporting of the presence of symptoms. Additionally, estimating prevalence of BPD in college samples is less often the sole focus of research, de-emphasizing the need for methodological rigour in diagnosis. Finally, the omission of key BPD criterion from validated scales may contribute to variability. Measures of BPD commonly tap high-risk behaviours, which raise concern about contagion and promotion of unsafe behaviours among students (e.g. self-harm). Resultantly, institutional review boards may require items are removed when participants are unidentifiable and cannot be appropriately referred.[13]

The characteristics of those surveyed may also represent a source of heterogeneity. For instance, while BPD manifests more commonly in female psychiatric samples, general population studies yield negligible difference in rates between genders;[9] however, it is unclear whether this trend replicates in college populations. Similarly, there are mixed findings relative to racial identification. For example, one earlier large-scale study suggests Hispanic people have lower rates of BPD, [14] but a comparative later large-scale study reported Hispanic people as having higher rates of BPD.[15] The aforementioned characteristics suggest that a systematic analysis of the literature pertaining to BPD in college populations, may serve to distinguish the contribution of methodology and study characteristics to variance in estimates of BPD prevalence between studies. Additionally, this undertaking may distinguish an overall pooled prevalence of the disorder in college populations, elucidate temporal trends, and identify student characteristics that have stronger associations with experiencing BPD symptoms.

Should the occurrence of clinically significant BPD symptoms be indicated as a prominent and growing health concern in college populations, this outcome may provide a foundation for the allocation of resources toward prevention and intervention within a college context. In turn, identifying student characteristics associated with the disorder could afford improved capacity to target resources toward students at higher risk of experiencing symptoms of BPD.

## Methods

### Search strategy

Literature was searched independently by two researchers employing the PRISMA Protocol[16] and Cochrane Guidelines.[17] In order to maximize both the statistical soundness of prevalence estimates and capture relevant studies, peer-reviewed publications dated from January 1994 to April 2014 were searched using fourteen electronic databases: AMED, Biological Abstracts, CINAHL Plus, Current Contents Connect, EBM Reviews, Embase, Google Scholar, Ovid MEDLINE, ProQuest Central, PsychINFO, PubMed, Scopus, Taylor & Francis Online and Web of Science (1998–2014; earliest accessible year was 1998). The search was limited from the year 1994 onward, to coincide with publication of the fourth edition of Diagnostic and Statistical Manual of Mental Disorders (DSM).[18] While BPD was first included in the DSM in 1980,[19] the wording, and number of criterion for BPD, differed to that of DSM-IV.[18] Measures of BPD predominantly reflect DSM criterion, which have remained unchanged over three subsequent editions of DSM.[12] Blashfield, Blum and Pfohl [20] demonstrated even minor changes to criterion result in considerable fluctuations of prevalence rates of personality disorders, thus we considered limiting the search may serve to ensure the construct under study, namely BPD, was reliably measured. The terms used in the searches varied according to the database utilized, and also included derivatives appropriate to variations in vernacular (i.e. college versus university). Predominantly, the search terminology employed was designed to capture the disorder, relevant population, and occurrence, thus included the terms: *Borderline Personality Disorder*, *college students*, *university students*, *prevalence*, *and symptoms or features*.

### Inclusion criterion

Reflective of the search terminology, and diagnostic characteristics of BPD as described above, inclusion was limited to studies that reported diagnostically relevant BPD, in college student populations, contained in peer reviewed studies published from January 1994 to April 2014. Further, the larger proportion of studies estimating BPD prevalence utilize self-report measures containing items that reflect either symptoms (subjective indications), or features (attributes) of BPD as opposed to diagnostic criterion.[21] Consequently, authors of the measures commonly caution the indicative rather than diagnostic interpretation of higher scores.[22] Nonetheless, measures of BPD predominantly report diagnostic cut-offs that vary considerably across measures. Subsequently, we only retained studies that either reported the percentage of participants within diagnostic range for BPD, or could be calculated as a proportion of the overall sample.

### Exclusion criterion

We excluded studies where arbitrary or dichotomous cut-off scores had been assigned, such as high BPD/low BPD. Where studies employed two levels of measurement, namely an initial self-report screen across a sample, followed by a structured interview for those that screened positive for BPD, we used the estimate from the self-report given the likelihood of inflated prevalence in those previously screened at interview stage. As the purpose of the review was to examine college populations, studies that examined other populations were excluded. Five of the studies reported on the same sample in two separate papers, thus we decided to retain the five studies containing greater methodological detail.

**Fig 1:**
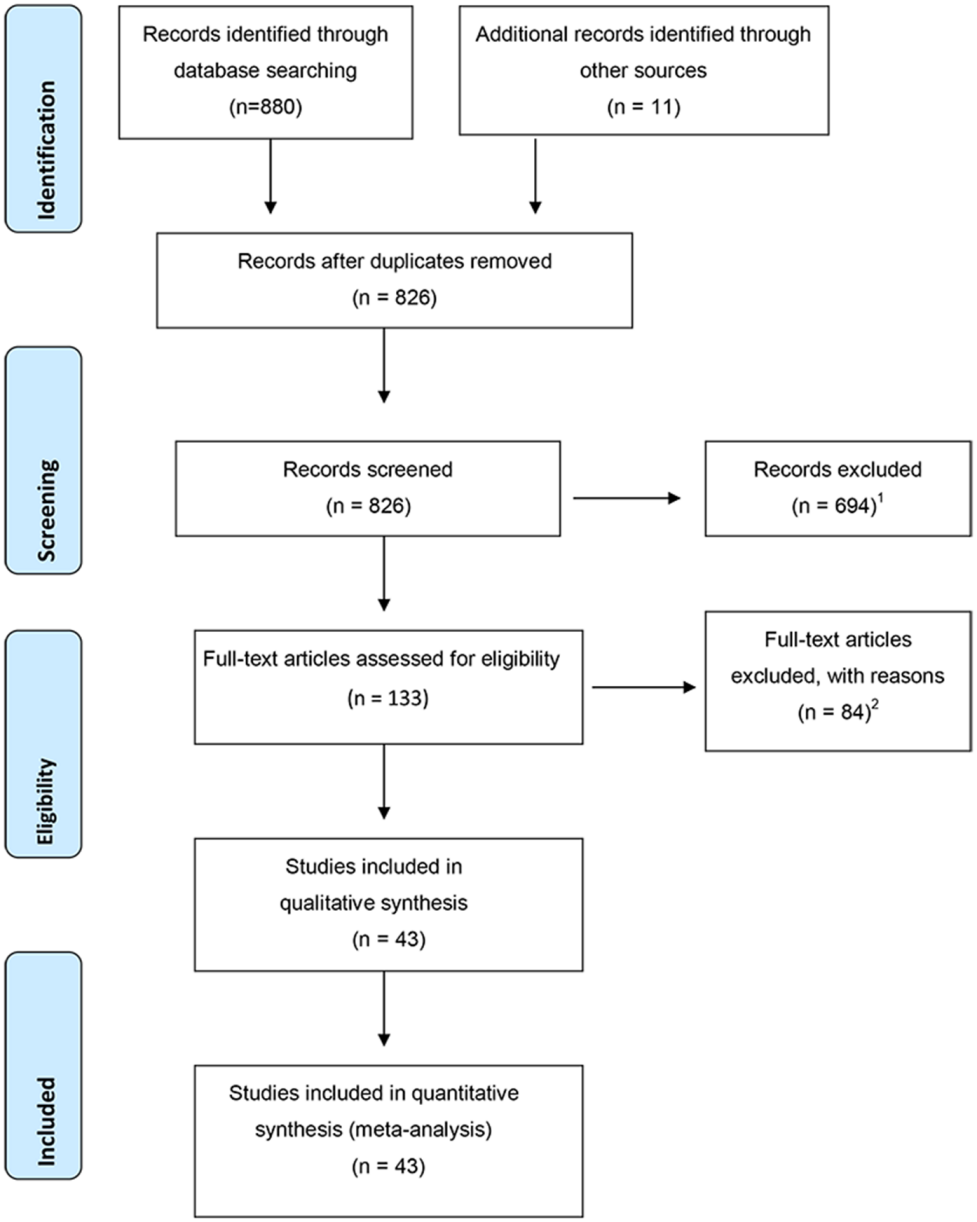
PRISMA flow diagram. ^1^Excluded due to studies sampling populations other than college students, or not having reported prevalence of BPD, or allowing calculable prevalence of BPD. ^2^As per exclusion Note 1, and also correspondence with authors.

The first database search retrieved 880 unique records, and 11 additional records found through other sources were also included. All records were subsequently screened by title, abstract, and full text, which resulted in 133 records subsequently undergoing examination. Resultant to this, 39 suitable articles were retained. Cited reference searches using author surname, initial, journal name and publication year resulted in no additional usable records. Hand searches of two journals that contained the greater proportion of suitable records (Journal of Personality Disorders, and Personality Disorders: Theory, Research and Treatment), retrieved 3 additional records; 4 more records known to the authors but not found in searches were added. Correspondence with authors resulted in the exclusion of 5 records due to methodological characteristics that falsely inflated prevalence (e.g. BPD cut-off changed to capture as low as three traits, and arbitrary or dichotomous cut-offs). Overall, this process (Fig 1) yielded 43 prevalence estimates from 43 records, which were retained in the analyses. The searches described above were replicated in July 2015 and did not yield any further suitable records (S1 and S2 Tables).

### Data extraction and coding

The data was extracted by the first author, and included characteristics of the population and study undertaken. This process was standardized using a data extraction form with parameters specified in the Cochrane Handbook,[23] then individual data items were checked against the corresponding data reported in the studies during two further separate examinations. In addition, the second author independently extracted the data from five of the 43 records, and also examined the data, and coding characteristics. The data for the five records extracted by the first and second author was compared across each item, and found to have 100% inter-rater agreement. The data extracted included: the prevalence of college students falling within the stated diagnostic range of BPD symptoms; the measure of BPD employed (Type of measure), publication year, data collection year, country, study level (e.g. undergraduate), mean age, gender, and racial characteristics of the sample. The methodological factors considered to account for variance between studies (moderator variables), included procedural characteristics encompassing participant anonymity, (yes/no), whether an incentive was offered for participation (yes/no), incentive type (course credit/cash/none), and primary research focus (BPD or other); the response rate, time period across which prevalence was assessed (e.g. week, month, lifetime), mode of measurement (e.g., interview or questionnaire), response format (e.g. yes/no, true/false or Likert), number of items in the measure, whether the measure reflected diagnostic traits or symptoms/features, and clinical cut-offs (numerical). Where information was unavailable the corresponding authors were contacted via email; 41.9% replied and subsequently there was 6.7% missing data overall.

### Statistical analysis

**Fig 2:**
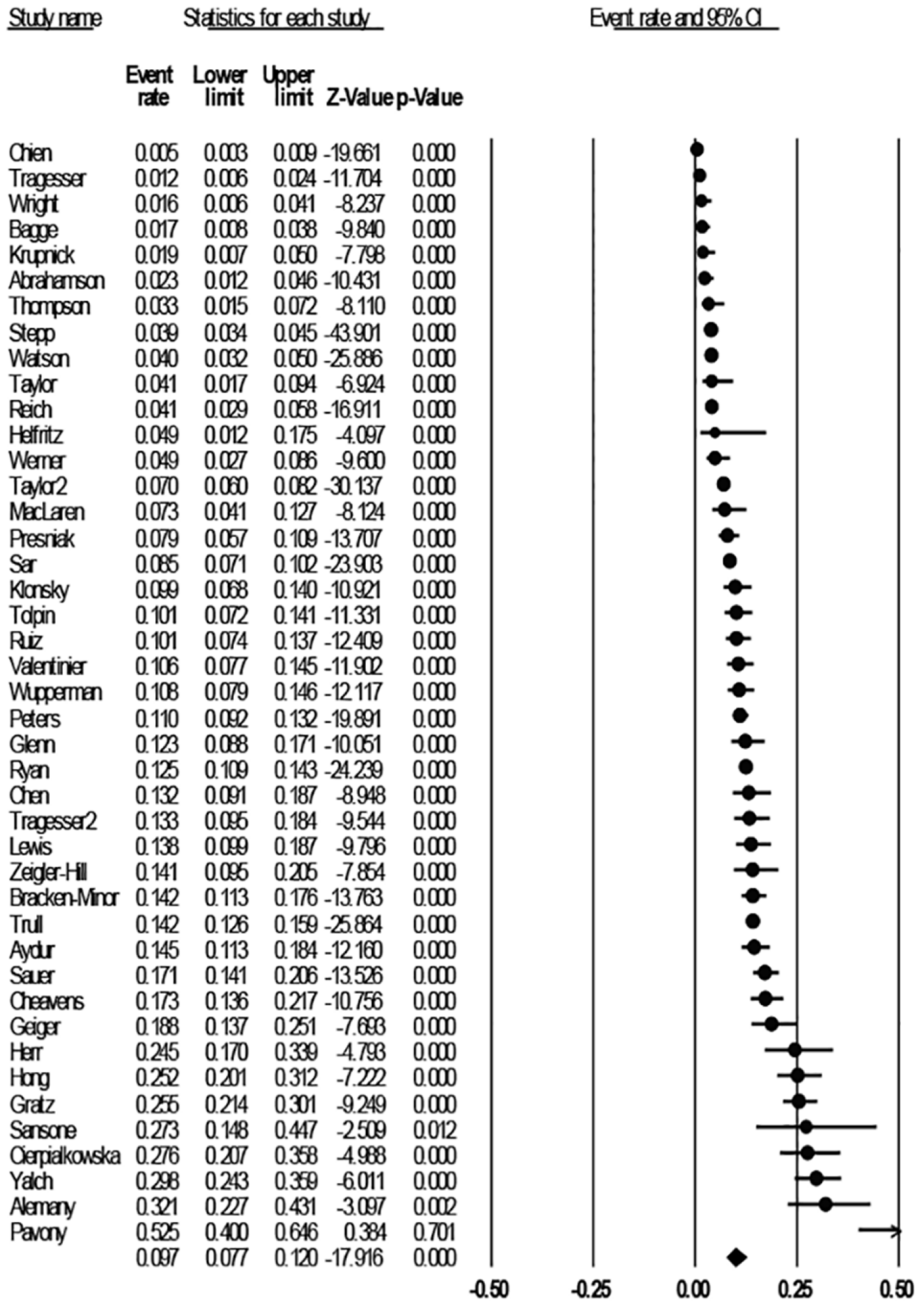
Studies included in the analysis sorted by prevalence.

Analyses were undertaken using Comprehensive Meta-Analysis, version 2.2.057.[24] The mean weighted event rate was estimated as a proportion (number of BPD cases/sample size). The calculations utilized a random effects model given the variability in BPD prevalence, sample characteristics across studies, and variances within studies. The studies were weighted by the inverse variance methods, and a random-effects model used to pool adjusted BPD prevalence at a 95% CI. The range of effects was assessed through a visual examination of the Forrest plot (Fig 2) showing the estimates and 95% CIs, and the weight of each point estimate.[24] Univariate meta-analyses were used to examine the influence of categorical moderator variables on pooled prevalence of BPD. The *I*^*2*^ value was calculated for each overall effect using Cochran’s (Q–df/Q) x 100%. [25,26] Thresholds for the interpretation of the *I*^2^ are reported to be contingent on both the magnitude and direction of effects, *p* ≤ .05, when a lower number of studies are examined. Higgins and Thompson [25] suggest *I*^2^ values of 0–40% might be considered as unimportant, 30–60% may represent moderate importance, 50–90% substantial importance, and 75–100% considerable heterogeneity.

Next, univariate meta-regression was conducted to examine the influence of the sample characteristics: mean age, gender, and racial composition, and study characteristics, namely, year published, clinical cut-offs, sample size, and country study was conducted in. The results were obtained from a mixed effects regression (Method of Moments), which calculates between-study τ^2^ (tau square) and compares this figure to the Z distribution.[27] Values of τ^2^ less than 1, taken in conjunction with a significant *p* value (*p* ≤ .05) are considered to represent significant heterogeneity.[26] In addition, publication bias was determined from a funnel plot, and Eggers test of the intercept to quantify any bias captured by the funnel plot, and test for significance across the studies.

## Results

### Study characteristics

The prevalence of BPD reported in the 43 included studies (see Fig 2) ranged from 0.5% to 32.1%, with an unadjusted lifetime prevalence of 9.7% (95% CI, 7.7–12.0; p < .005). The total number of participants was 26,343 (range: 33–5000), represented predominantly by participants in the USA (n = 36, 86.1%), followed by Canada (n = 3, 4.7%), then Spain, Poland, Taiwan, and Turkey at one study each. Over the 20-year period there was an increase in the number of publications reporting clinically significant BPD estimates in college populations, with six articles published between 1994 and 2000, 10 between 2001 and 2007, and 27 between 2008 and 2014. Overall, 66.7% (n = 28) of studies comprised of research focused primarily on BPD, followed by 6.7% (n = 3) focused on non-suicidal self-injury (NSSI). The average time from completion of data collection to publication was 4.3 years (data available for n = 26 studies). Participant age ranged from 17 to 66 years, with a mean age of 19.4 (*SD* = 1.4). Of the 43 records, 40 (93%) sampled both genders, and three (7%) comprised of females only. Overall, females represented 64.7% (n = 17,044) of the combined sample. Collectively, 40 of the studies sampled undergraduates, one study sampled postgraduates and two studies sampled both groups. Participant race was predominantly comprised of those identifying as White/Caucasian (68.1%, n = 17,940), followed by ‘other race’ (11.7%, n = 3082), Asian (8.7%, n = 2292), African American (7.7%, n = 2,028), and Hispanic (3.8%, n = 1001). Participant responses were anonymous in 30 (68.9%) of the studies; 37 (86.6%) of studies offered an incentive, most commonly course credit (87.2%).

BPD was measured using 13 tools across the studies included. Of the tools, five (11.6%, N = 43) studies employed structured clinical interviews, predominantly represented by the Structured Clinical Interview for DSM-III-R / DSM-IV Personality Disorders (SCID-II),[28] which was used in two studies. Of the 30 studies utilizing self-report measures, the PAI-BOR [22] predominated (48.8%, n = 15), followed by the McLean Screening Instrument for Borderline Personality Disorder [29] (MSI-BPD; 11.6%, n = 3). While the structured clinical interviews all used DSM-IV traits as items, the self-report measures employed in 30 studies primarily utilized BPD features or attributes[21] (65.5%, n = 19), followed by symptoms (subjective indication;[21] 33.3%, n = 10), and the remaining measure used DSM traits. When considering response format of self-report items, 51.2% used a 4-point Likert scale reflecting level of agreement with statements, and of these, 74.4% measured the veracity of statements reflecting characteristics of the person (e.g. true/false), followed by frequency of symptoms (11.6%), then presence of symptoms or personal characteristics (i.e. yes/no; 9.3%), and finally severity of listed symptoms (4.7%). Prevalence was measured across the lifetime in 41 studies, over a month in one study, and over a two-week period in the remaining study. The number of items in the measures ranged from 3 to 140, and the measures had not been altered in all but one study, whereby a single item relating to self-harm had been removed prior to administering the questionnaire, in order to comply with ethical committee directives.

### Pooled prevalence of BPD in college populations and changes over time

Prevalence ranged from 0.5% to 32.1% across the studies, with an unadjusted lifetime prevalence of 9.7% (95% CI, 7.7–12.0; *p <* .005), *I*^*2*^ = 96.2. The analyses were re-run omitting the studies representing extreme values, however this did not significantly influence the overall prevalence rates or between study heterogeneity (i.e. Pavony[30]omitted: 9.2%, 95% CI 7.4–11.4, *I*^*2*^ = 96.0 *p* < .005; Chien [31] omitted: 10.4%, 95% CI 8.4–12.7, *p <* .005, *I*^*2*^ = 95.8). The prevalence of BPD varied over time: 7.8% (95% CI 4.2–13.9) between 1994 and 2000; 6.5% (95% CI 4.0–10.5) during 2001 to 2007; and 11.6% (95% CI 8.8–15.1) from 2008 to 2014, however heterogeneity across time was not significant (*p =* .09, *I*^*2*^ = 72.6).

### Methodological factors contributing to between-study heterogeneity

Univariate meta-analyses were used to assess the influence of methodological factors on reported prevalence rates (Table 1). Overall the *I*^*2*^ statistic ranged from 37.5 to 94.6%; anonymity, incentive type, focus of the research, and participant type were indicative of considerable heterogeneity at *p* < .05. In the initial analysis, the type of measure was not associated with heterogeneity (*p* = .34). However of the 13 measures, eight were only used once, thus the analysis was re-run omitting these lone items. Subsequently, the type of measure was associated with heterogeneity of substantial importance.[26] In detail, studies that provided anonymity in responses, offered course credit as an incentive, were focused on the topic of BPD, sampled postgraduates, and utilized the International Personality Disorder Examination (IPDE),[32] were associated with higher rates of BPD.

**Table 1:** Pooled Prevalence Estimates and Proportion of Variance Explained by Methodological Factors (N = 43).

	Overall Effect size			Between Study Heterogeneity			
Category^2^	Pooled prev %	95% CI	*Z*^1^	Cochran Q	df (Q)	*p*	*I*^*2*^%
**Anonymity**	**7.9**	**2.9–20.1**	-4.5***	**18.6**	**1**	**.000**	**94.6**
Yes (n = 30)	12.8	10.2–16.0	-14.4***				
No (n = 13)	4.7	3.1–7.0	-14.1***				
**Incentive type**	**5.4**	**1.8–14.8**	-5.0***	**19.1**	**2**	**.000**	**90.1**
Course credit (n = 34)	12.1	9.6–15.3	-14.6***				
None (n = 5)	3.9	2.0–7.6	-8.9***				
Cash (n = 4)	2.7	1.1–6.5	-7.7***				
**Focus of research**	**9.0**	**5.3–14.7**	-8.0***	**4.6**	**1**	**.032**	**78.6**
BPD (n = 29)	11.4	8.7–14.7	-13.6***				
Other (n = 14)	6.7	4.4–10.0	-11.8***				
**Participant type**	**17.6**	**6.2–40.9**	-2.6**	**8.1**	**2**	**.017**	**75.3**
Postgraduates (PG; n = 1)	32.1	8.9–69.5	-0.9*				
UG & PG (n = 2)	25.4	10.0–50.7	-1.9				
Undergraduates (UG; n = 40)	8.9	7.1–11.1	-18.3***				
**Data collection format**^3^	7.9	3.9–15.3	-6.4***	3.7	1	.054	73.0
**Incentive**^4^	8.3	4.6–14.5	-7.5***	3.2	1	.072	68.8
**Type of Measure**^5^	**9.4**	**5.9–14.6**	-8.7***	**27.5**	**13**	**.011**	**52.7**
IPDE^6^ (n = 4)	21.6	17.0–27.0	-4.8***				
MSI-BPD^7^ (n = 5)	13.6	7.4–23.7	-5.3***				
PAI-BOR^8^ (n = 20)	9.3	6.8–12.7	-12.9***				
**Construct measured**^9^	8.2	4.9–13.5	-8.5***	5.7	3	.129	47.4
**Measure format**^10^	10.1	7.4–13.6	-12.7***	2.8	4	.591	42.9
**Criterion measured**^11^	9.4	6.5–13.3	-11.3***	3.2	2	.198	37.5
**Time period** ^12^	11.5	5.8–21.6	-5.4***	2.3	2	.318	30.0

*Note*.

* p < .05

** p < .01

*** p < .001

^1^ Random effects analysis reported, ranked by *I*^2^

^2^ Only categories with significant heterogeneity (bold) have sub-levels reported (in italics)

^3^ Self-report or clinical interview

^4^ Incentive: yes/no

^5^ Type of measure only reported where measure n ≥4

^6^ The International Personality Disorder Examination

^7^ McLean Screening Instrument for Borderline Personality Disorder

^8^ Personality Assessment Inventory, Borderline Features Scale

^9^ Features, symptoms or traits of BPD

^10^ 3,4 or 5-point scale, true/false or yes/no

^11^ Frequency, presence, severity or veracity (true/false) of BPD items

^12^ One month, 14 days, or life

### Study or sample characteristics contributing to between study heterogeneity

In univariate meta-regressions heterogeneity was apparent across all the variables with τ^2^ ranging from .407 to .635 (Table 2). Studies with a smaller sample size had a lower number of participants with BPD, while participants who identified as Asian also reported lower rates of BPD. Alternatively participants identified in the “other” racial category were more likely to warrant a diagnosis of BPD.

**Table 2:** Results of univariate meta-regression^1^.

Variable	Category 1	Category 2	Point	Standard	95% CI	*Z*	τ^2^
	(*k*, *N*)^2^	(*k*, *N*)	estimate	error			
Country	USA = 0	Other = 1	-.132	.115	-.358; .094	-1.147	.624
	(37, 22681)	(6, 3662)					
Year of publication	1994–2014		.034	.024	-.013; .080	1.420	.626
	(43, 26343)						
Clinical cut-offs	4–70		-.001	.007	-.014; .014	-.001	.634
	(43, 26343)						
**Sample size**^3^	**33–5000**		**-.001**	**.001**	**-.001; -.000**	-3.835***	**.407**
	**(43, 26343)**						
M Age, years	18–30		-.019	.023	-.064; .026	-.821	.610
	(40, 25670)						
Female%	37–100		.015	.010	-.004; .034	1.570	.630
	(43, 17044)						
Male%	0–63		-.015	.010	-.034; .004	-1.571	.630
	(43, 9299)						
White/Caucasian%	0–94		-.002	.004	-.010; .006	-.530	.631
	(37,17940)						
Black/African%	0–37.1		-.005	.014	-.033; .023	-.352	.632
	(36, 2028)						
Hispanic/Latin%	0–14		.025	.032	-.038; .088	.768	.635
	(36, 1001)						
**Asian%**^3^	**0–100**		**-.018**	**.007**	**-.032; .005**	-2.61**	**.601**
	**(36, 2292)**						
**Other%**^3^	**0–100**		**.018**	**.007**	**.003; .032**	2.42*	**.564**
	**(36, 3082)**						

*Note*.

* p < .05

** p < .01

*** p < .001

^1^ Results from Mixed effects regression (Method of Moments)

^2^ Significant (p < .05) results shown in bold

^3^ *k* = number of studies; *N* = total sample size

## Discussion

We aimed to measure methodological characteristics that contribute to heterogeneity across estimates of BPD in college populations reported in the literature, to establish pooled prevalence, ascertain whether rates had changed over time, and identify at risk groups in terms of demographic characteristics. Methodological factors that accounted for considerable heterogeneity between estimates of BPD in college student populations were: anonymity, incentive type, focus of the research, and participant type. While the type of measure (e.g. PAI-BOR) [22] employed had substantial importance toward influencing between-study heterogeneity. The characteristics of the sample that contributed to significant heterogeneity between studies were sample size, and identifying as Asian or “other” race.

In the context of BPD, anonymity of responses may be particularly influential given criterion includes behaviors with low social desirability, and implications for participant safety. Specifically, endorsement of criterion relating to suicidal ideation and attempts is associated with lower response rates, as the behavior is highly stigmatized, [33] and may trigger a duty of care whereby researchers are ethically required to contact and refer participants.[34] Similarly, shame is a common feature in those with BPD, which may also act as motivator to under-report problematic behaviors such as aggressive outbursts, or substance use, when the person is identifiable.[35]

The type of incentive offered was associated with unique heterogeneous influence on prevalence rates, with course credit associated with studies that reported higher rates of BPD. While offering incentives has been reported to bear no effect toward bias in sample demographics,[36] incentives such as course credit may be particularly attractive to college students, even more so than cash. In turn, studies with larger samples reported higher rates of BPD, representing a well-documented relationship between the increase in probability of attracting higher rates of any construct measured when more people are sampled.[37] Nonetheless, prevalence of BPD has been shown to be lower in age-matched general population samples (e.g. 3.2%); [38] which suggests that a pooled prevalence of 9.7% indicates BPD traits may be apparent in college populations.

The topic or construct under study played a role in variations of prevalence, specifically, studies focused on BPD had a significantly higher prevalence (11.4%), compared to those that did not (6.7%). Participants are attracted to studies that are either interesting or relevant to them,[39] and as psychology students were sampled in 74% of the studies analyzed, interest in personality disorders may be more apparent when compared to other study disciplines. Alternatively, the finding on participant type should be interpreted with caution as only one study utilized a solely postgraduate sample, and two studies both undergraduates and postgraduates. BPD symptom frequency and severity is thought to decrease as the person matures,[40] yet the current study suggests the inverse of this relationship, which may be related to there being only 49 postgraduates, and 492 combined study level participants analyzed. Similarly, the finding that the type of measure employed was a source of heterogeneity should be interpreted with considerable caution. The IPDE [30] was associated with a notably high prevalence rate of 21.6%, which was largely accounted for by the Alemany Martinez, Aytés and Escoda study.[8] The aforementioned study examined personality disorder characteristics as one of a multitude of factors that may have a relationship with burnout in dentistry students.[8] The authors had cautioned that diagnosis was not a function of the study, and as such, methodological rigor in establishing those above the clinical cut-offs on the IPDE may not have been emphasized.

The finding for racial categories may be influenced by the fact that USA-based studies predominated in the review. In addition, 75.7% of these studies contained “other” racial categories ranging from 0.6 to 30% of the sample. Participant race was not the focus of the research in any of the studies reviewed; however in the few cases where the “other” category was distinguished, it largely contained Native Americans. This group is strongly under-represented in US college populations; yet tend to report higher rates of BPD (e.g. 5.0%).[6] Notwithstanding, the “other” race category represented 11.7% of all participants in the review, and was associated with higher BPD prevalence, emphasizing the value of greater delineation of racial groups in research to allow meaningful interpretation of groups with higher risk of the disorder.

Alternatively, there has been consistent evidence that people who identify as Asian within US samples report lower rates of BPD,[6,41] and the results of the current review lend support to this characteristic. Similarly, that no difference was found between genders in rates of BPD is consistent with a range of literature.[9,42] In college populations, as with age-matched community samples, it would appear that both males and females are equally likely to report traits of the disorder. Nonetheless, this may be because college men report more impulsive or substance use behaviors represented in measures of BPD, as opposed to manifesting the disorder. Finally, the results of the current study were unable to elucidate whether the prevalence of BPD in college populations has increased over time. A similar lack of distinction is apparent in the literature for community samples,[43] however as the current study is the first of its type, replication may assist in distinguishing temporal trends.

### Limitations

Several factors suggest that the results should be interpreted with caution. With reference to pooled prevalence, sample size was a predictor of heterogeneity, and samples included in the current study ranged from as low as 33, to as high as 5000 participants. Similarly, there was considerable variance in prevalence estimates ranging from 0.5 to 32.1%. While every attempt was made to be comprehensive, it is possible that variations in statistical analyses, methodological issues, or data manipulation not assessed in the current study, may have accounted for some of the variance.

An additional limitation pertains to how generalizable the results of the review may be. Across the literature, females, undergraduates, and Caucasians tend to be over-represented in college samples in research, [44] and this effect may be increased in systematic reviews due to the magnification of skewed populations when analysed.[25] Relatively recent US college enrolment figures indicate females comprise 53.6% of all US college enrolments, while Caucasians represent 76%, and postgraduates 12.6%. [45] In the US studies in the review, females represented 70.3%, Caucasians 79.2% and postgraduates 2.3%, indicating that females and Caucasians were over-represented while postgraduates were significantly under-represented in the current sample.

## Conclusions

The findings of the study suggest important considerations, and recommendations for future research. First, anonymity has an important role in methodology employed to assess for BPD. People with BPD are characteristically proactive in help seeking when experiencing suicidal ideation,[46] which may offset concerns of identifying at-risk participants, when compared with the utility of ascertaining at risk groups.

Consistent with previous findings,[29,47] self-report measures were not associated with significant heterogeneity when compared with clinical interviews, thus larger scale studies could employ freely available, empirically validated self-report measures such as the MSI-BPD, [29] to reduce the time and cost of the research.

There is a clear need for research focusing on BPD in college students to be conducted in countries other than the US. The systematic searches in the current study failed to uncover reported prevalence in the United Kingdom, Australia, and the greater proportion of Europe and Asia. While US-based studies are unquestionably useful, factors that are associated with variance such as race cannot be generalized to countries where college students tend to be more homogenous. Relatedly, a range of demographic characteristics associated with BPD was not measured in the review. Low socioeconomic status, being single or divorced, and identifying as homosexual have all been associated with a diagnosis of BPD.[4,5] This information was not available for a large proportion of the studies, and may be worth including in future research.

The review elucidated the need for consistency in measurement across studies. While not a source of heterogeneity, the items, and resulting constructs used to measure BPD were various and diverse, such as traits versus symptoms or features. Relatedly, measurement of BPD over short time frames, or cross-sectional studies may result in prevalence underestimation given the level of lability associated with BPD symptoms. As such, directions given to participants when responding should specify that they reflect on the presence of symptoms over the previous year, at minimum, in order to assess for pervasive patterns of behavior characteristic to personality disorders.

Finally, the review suggests resource allocation considerations for colleges. At a pooled prevalence rate of close to 10% the findings suggest that BPD is apparent in college student populations. Given symptoms of the disorder include high-risk behaviours such as self-harm, suicidal expression and aggression; the study findings have particular relevance for college-based mental health services. Within an Australian context, recent federal funding cuts to the college sector have resulted in retractions of perceived non-essential services (including counseling services)[48,49] suggesting mental health staff are required to allocate limited resources with greater efficiency. In turn, college-based treatment programs such as modified Dialectical Behaviour Therapy, have demonstrated promising results in treating students with BPD symptoms cost-effectively.[50]

In sum, 47% of the heterogeneity observed in BPD estimates within college populations was due to either methodological or sample-related factors. Wherever possible, standardization across studies would significantly assist in improving the reliability of future reviews.

## Supporting Information

S1 Table PRISMA checklist. (DOC)

S2 Table Complete Appendices. (DOCX)
