# Supplementary material for: Prevalence of Borderline Personality Disorder in University Samples: Systematic Review, Meta-Analysis and Meta-Regression
Source: PLoS One. 2016 May 12;11(5):e0155439. doi: 10.1371/journal.pone.0155439 (PMC4865108; doi:10.1371/journal.pone.0155439)
Supplement: S2 Table — (DOCX) [file pone.0155439.s002.docx]

Appendix 2A: Database Search Histories (Search 1)

**AMED** (Allied and Complementary Medicine)

| **No** | **Query Results** | **Results** | **Date** |
| --- | --- | --- | --- |
| #9 | #2 AND #6 | 5 | 31.3.14 |
| #8 | #2 AND #4 | 0 | 31.3.14 |
| #7 | #2 AND #4 AND #6 | 0 | 31.3.14 |
| #6 | ''prevalence or occurrence or frequency or features” (date 1994-2014) | 9,491 | 31.3.14 |
| #4 | “college students or university students or undergraduates or postgraduates or pupils” (date 1994-2014) | 708 | 31.3.14 |
| #2 | “borderline personality disorder or BPD or borderline personality” (date 1994-2014) | 58 | 31.3.14 |

**Biological Abstracts**

| **Search**  **ID#** | **Search Terms** | **Search Options** | **Actions** | **Date** |
| --- | --- | --- | --- | --- |
| S11 | S9 AND S10 | **Search modes** - Boolean/Phrase | 0 | 31.03.14 |
| S10 | S6 AND S8 | **Search modes** - Boolean/Phrase | 65,812 | 31.03.14 |
| S9 | S2 AND S4 | **Search modes** - Boolean/Phrase | 9 | 31.03.14 |
| S8 | “traits OR symptoms OR characteristics OR features”  (1994-2014; English) | **Search modes** - Boolean/Phrase | 540,953 | 31.03.14 |
| S6 | “prevalence OR occurrence OR frequency"(1994-2014; English) | **Search modes** - Boolean/Phrase | 511,852 | 31.03.14 |
| S4 | “college students OR university students OR undergraduate OR postgraduate OR pupil"(1994-2014; English) | **Search modes** - Boolean/Phrase | 6,371 | 31.03.14 |
| S2 | “borderline personality disorder OR BPD OR borderline personality"  (1994-2014; English) | **Search modes** - Boolean/Phrase | 2,721 | 31.03.14 |

**CINAHL Plus**

| **Search**  **ID#** | **Search Terms** | **Search Options** | **Actions** | **Date** |
| --- | --- | --- | --- | --- |
| S7 | S5 AND S6 | **Search modes** - Boolean/Phrase | 2 | 30.03.14 |
| S6 | S3 AND S4 | **Search modes** - Boolean/Phrase | 26,614 | 30.03.14 |
| S5 | S1 AND S2 | **Search modes** - Boolean/Phrase | 33 | 30.03.14 |
| S4 | “traits OR symptoms OR characteristics OR features”  (1994-2014; English) | **Search modes** - Boolean/Phrase | 209,362 | 30.03.14 |
| S3 | “prevalence OR occurrence OR frequency"(1994-2014; English) | **Search modes** - Boolean/Phrase | 142,815 | 30.03.14 |
| S2 | “college students OR university students OR undergraduate OR postgraduate OR pupils"(1994-2014; English) | **Search modes** - Boolean/Phrase | 21,069 | 30.03.14 |
| S1 | “borderline personality disorder OR BPD OR borderline personality"  (1994-2014; English) | **Search modes** - Boolean/Phrase | 2,444 | 30.03.14 |

**Current contents connect (through Web of Science)**

| **Search**  **ID#** | **Search Terms** | **Search Options** | **Actions** | **Date** |
| --- | --- | --- | --- | --- |
| S3 | AND **TOPIC:** (prevalence OR occurrence OR frequency) | **Search modes** - Boolean/Phrase | 48 | 7.04.14 |
| S2 | **Refined by:** **TOPIC:** (college students OR university students OR undergraduates OR postgraduates or pupils) | **Search modes** - Boolean/Phrase | 222 | 7.04.14 |
| S1 | “**TOPIC:** (borderline personality disorder OR BPD OR borderline personality)  **Timespan:** 1998-2014. **Indexes:** ABES, SBS, CM, LS, PCES, ECT, AH, BC, EC. “ | **Search modes** - Boolean/Phrase | 8,138 | 7.04.14 |

**EBM reviews** **(Allied and Complementary Medicine)**

| **No** | **Query Results** | **Results** | **Date** |
| --- | --- | --- | --- |
| #11 | #3 AND #6 | 4 | 20.04.14 |
| #10 | #3 AND #6 AND #9 | 2 | 20.04.14 |
| #9 | ''prevalence or occurrence or frequency” (date 1994-2014) limit 1 to english language [Limit not valid in CDSR,ACP Journal Club,DARE,CCTR,CLCMR; records were retained] | 48698 | 20.04.14 |
| #6 | “college students or university students or undergraduates or postgraduates or pupils” (date 1994-2014) limit 1 to english language [Limit not valid in CDSR,ACP Journal Club,DARE,CCTR,CLCMR; records were retained] | 1832 | 20.04.14 |
| #3 | “borderline personality disorder or BPD or borderline personality” (date 1994-2014)  limit 1 to english language [Limit not valid in CDSR,ACP Journal Club,DARE,CCTR,CLCMR; records were retained] | 669 | 20.04.14 |

**Embase**

| **No** | **Query Results** | **Results** | **Date** |
| --- | --- | --- | --- |
| #5 | #2 AND #3 AND #4 | 27 | 20.04.14 |
| #4 | prevalence OR occurrence OR frequency | 1,532,696 | 20.04.14 |
| #3 | college AND students OR university AND students OR undergraduates OR postgraduates OR pupils | 129,414 | 20.04.14 |
| #1 | Keyword search **"borderline personality disorder OR BPD or borderline personality"[MeSH Major Topic]** (date 1994-2014; English) | 336 | 21.04.14 |

**Google Scholar**

| **No** | **Query Results** | **Results** | **Date** |
| --- | --- | --- | --- |
| #2 | Date 1994-2014 | 129 | 20.04.14 |
| #1 | borderline personality disorder or borderline personality or bpd and college students or university students or undergraduates and postgraduates and prevalence | 236 | 20.04.14 |

**Ovid MEDLINE Search (PubMed)**

| **Search** | **Most Recent Queries** | **Date** | **Result** |
| --- | --- | --- | --- |
| #10 | Search **#4 AND #9** (date 1994-2014) | 25.03.14 | 8 |
| #9 | Search **#2 AND #6** (date 1994-2014) | 25.03.14 | 847 |
| #8 | Search **traits OR symptoms OR characteristics OR features**(date 1994-2014) | 25.03.14 | 815452 |
| #6 | Search “**prevalence OR occurrence OR frequency”** (date 1994-2014; English) | 25.03.14 | 764581 |
| #4 | Keyword search **"college students OR university students OR undergraduates OR postgraduates OR pupils"[MeSH Major Topic]** (date 1994-2014, English) | 25.03.14 | 18184 |
| #2 | Keyword search **"borderline personality disorder OR BPD or borderline personality"[MeSH Major Topic]** (date 1994-2014; English) | 25.03.14 | 6122 |

**Proquest Central**

| **Search** | **Most Recent Queries** | **Date** | **Result** |
| --- | --- | --- | --- |
| #7 | (((((((((borderline personality disorder) OR (bpd OR borderline personality)) AND (college students OR university students OR undergraduates OR postgraduates)) AND (prevalence OR occurrence)) AND (traits OR symptoms OR characteristics OR features)) NOT adolescent) NOT patient) NOT inmates) NOT inpatients) NOT school | 21.4.14 | 118 |
| #6 | ((((((borderline personality disorder) OR (bpd OR borderline personality)) AND (college students OR university students OR undergraduates OR postgraduates)) AND (prevalence OR occurrence)) AND (traits OR symptoms OR characteristics OR features)) NOT adolescent) NOT patient | 21.4.14 | 325 |
| #5 | (((((borderline personality disorder) OR (bpd OR borderline personality)) AND (college students OR university students OR undergraduates OR postgraduates)) AND (prevalence OR occurrence)) AND (traits OR symptoms OR characteristics **OR features**)) NOT adolescent | 21.4.14 | 1,391 |
| #4 | **((((borderline personality disorder) OR (bpd OR borderline personality)) AND (college students OR university students OR undergraduates OR postgraduates)) AND (prevalence OR occurrence)) AND (traits OR symptoms OR characteristics OR** features**)** (date 1994-2014) | 21.4.14 | 3,876 |
| #3 | **(((borderline personality disorder) OR (bpd OR borderline personality)) AND (college students OR university students OR undergraduates OR postgraduates)) AND (prevalence OR occurrence)** (date 1994-2014; English) | 21.4.14 | 4,063 |
| #2 | ((borderline personality disorder) OR (bpd OR borderline personality)) AND (college students OR university students OR undergraduates OR postgraduates) | 21.4.14 | 8,370 |
| #1 | Keyword search **"borderline personality disorder OR BPD or borderline personality"[MeSH Major Topic]** (date 1994-2014; English) | 21.4.14 | 24,228 |

**PsycINFO Search**

| Search  ID# | **Search Terms** | **Actions** | **Date** |
| --- | --- | --- | --- |
| #11 | #9 AND #10 | 0 | 26.03.14 |
| #10 | #6 AND #9 | 16 | 26.03.14 |
| #9 | #2 AND #4 | 119 | 26.03.14 |
| #8 | “traits or symptoms or characteristics or features” | 312716 | 26.03.14 |
| #6 | “prevalence or occurrence or frequency” **(1994-2014; English)** | 220181 | 26.03.14 |
| #4 | “college students or university students or undergraduates or postgraduates or pupils” **(1994-2014; English)** | 76351 | 26.03.14 |
| #2 | “borderline personality disorder OR BPD or borderline personality" **(1994-2014; English)** | 6349 | 26.03.14 |

**PubMed**

| Search  ID# | **Search Terms** | **Actions** | **Date** |
| --- | --- | --- | --- |
| 4 | 1 AND 2 AND 3 **(1994-2014)** | 44 | 23.4.14 |
| 3 | (("epidemiology"[Subheading] OR "epidemiology"[All Fields] OR "prevalence"[All Fields] OR "prevalence"[MeSH Terms]) OR ("epidemiology"[Subheading] OR "epidemiology"[All Fields] OR "incidence"[All Fields] OR "incidence"[MeSH Terms])) OR ("epidemiology"[Subheading] OR "epidemiology"[All Fields] OR "occurrence"[All Fields] OR "epidemiology"[MeSH Terms] OR "occurrence"[All Fields]) AND ("1994/01/01"[PDAT] : "2014/12/31"[PDAT]) | 1,720,137 | 23.4.14 |
| 2 | (((college[All Fields] AND ("students"[MeSH Terms] OR "students"[All Fields])) OR (("universities"[MeSH Terms] OR "universities"[All Fields] OR "university"[All Fields]) AND ("students"[MeSH Terms] OR "students"[All Fields]))) OR undergraduate[All Fields]) AND postgraduate[All Fields] AND ("1994/01/01"[PDAT] : "2014/12/31"[PDAT]) | 2,166 | 23.4.14 |
| 1 | (("borderline personality disorder"[MeSH Terms] OR ("borderline"[All Fields] AND "personality"[All Fields] AND "disorder"[All Fields]) OR "borderline personality disorder"[All Fields]) OR bpd[All Fields]) OR ("borderline personality disorder"[MeSH Terms] OR ("borderline"[All Fields] AND "personality"[All Fields] AND "disorder"[All Fields]) OR "borderline personality disorder"[All Fields] OR ("borderline"[All Fields] AND "personality"[All Fields]) OR "borderline personality"[All Fields]) AND ("1994/01/01"[PDAT] : "2014/12/31"[PDAT]) | 8,261 | 23.4.14 |

**Scopus**

| Search  ID# | **Search Terms** | **Actions** | **Date** |
| --- | --- | --- | --- |
| 4 | 1 AND 2 AND 3 **>1993** | 11 | 28.04.14 |
| 3 | TITLE-ABS-KEY(prevalence OR occurrence OR frequency) AND PUBYEAR > 1993 | 2,431,047 | 28.04.14 |
| 2 | TITLE-ABS-KEY(university students OR college students OR undergraduates OR postgraduates) AND PUBYEAR > 1993 | 120,170 | 28.04.14 |
| 1 | TITLE-ABS-KEY (borderline personality disorder OR bpd OR borderline personality) (1994-2014) | 7403 | 28.04.14 |

**Taylor & Francis online**

| Search  ID# | **Search Terms** | **Actions** | **Date** |
| --- | --- | --- | --- |
| 4 | 1 AND 2 AND 3 (1994-2014) | 20 | 28.04.14 |
| 3 | Search Everything(prevalence OR occurrence OR frequency) AND PUBYEAR > 1993 | 559,054 | 28.04.14 |
| 2 | Search Everything (university students OR college students OR undergraduates OR postgraduates) AND PUBYEAR (1994-2014) | 969,797 | 28.04.14 |
| 1 | Search Everything (borderline personality disorder OR bpd OR borderline personality) (1994-2014) | 540 | 28.04.14 |

**Web of Science**

| Search ID# | **Search Terms** | **Actions** | **Date** |
| --- | --- | --- | --- |
| S3 | Refined by: **TOPIC:** *(college students or university students or undergraduates or postgraduates or pupils)* AND **TOPIC:** *(prevalence OR occurrence OR frequency)*  Timespan=1994-2014. Indexes=SCI-EXPANDED, SSCI, A&HCI, CPCI-S, CPCI-SSH, BKCI-S, BKCI-SSH, CCR-EXPANDED, IC. | 55 | 26.3.14 |
| S2 | *(borderline personality disorder OR BPD or borderline personality)*  Refined by: **TOPIC:** *(college students or university students or undergraduates or postgraduates or pupils)*  Timespan=1994-2014. Indexes=SCI-EXPANDED, SSCI, A&HCI, CPCI-S, CPCI-SSH, BKCI-S, BKCI-SSH, CCR-EXPANDED, IC. | 250 | 26.3.14 |
| S1 | *(borderline personality disorder OR BPD or borderline personality)*  Timespan=1994-2014. Indexes=SCI-EXPANDED, SSCI, A&HCI, CPCI-S, CPCI-SSH, BKCI-S, BKCI-SSH, CCR-EXPANDED, IC. | 11,098 | 26.3.14 |

**Other databases searched:**

| **Database** | **Date Searched** | **Records per total search terms** |
| --- | --- | --- |
| AEI : Australian Education Index | 31.3.14 | 0 |
| AMI : Australasian medical index | 31.3.14 | 3 (0 relevant) |
| APAIS : Australian public affairs information service | 31.3.14 | 13 (0 relevant) |
| BMJ Best Practice | 1.4.14 | 117 (0 relevant) |
| Cochrane Library | 1.4.14 | 6/8409 (0 relevant) |
| MIMS Online | 20.4.14 | 0 |
| Informit Online | 21.4.14 | 209 (0 relevant) |

**Database Search Histories: Search 2; 29-30^th^ July 2015.**

**AMED** (Allied and Complementary Medicine)

| **No** | **Query Results** | **Results** | **Date** |
| --- | --- | --- | --- |
| #9 | #2 AND #6 | 7 | 29.7.2015 |
| #8 | #2 AND #4 | 0 |  |
| #7 | #2 AND #4 AND #6 | 0 |  |
| #6 | ''prevalence or occurrence or frequency or features” (date 1980-2014) | 12657 |  |
| #4 | “college students or university students or undergraduates or postgraduates or pupils” (date 1980-2014) | 804 |  |
| #2 | “borderline personality disorder or BPD or borderline personality” (date 1980-2014) | 78 |  |

**None usable or new**

**Biological Abstracts**

| **Search**  **ID#** | **Search Terms** | **Search Options** | **Actions** | **Date** |
| --- | --- | --- | --- | --- |
| S11 | S9 AND S10 | **Search modes** - Boolean/Phrase | **1** | 30.7.15 |
| S10 | S6 AND S8 | **Search modes** - Boolean/Phrase | 123792 |  |
| S9 | S2 AND S4 | **Search modes** - Boolean/Phrase | **23** |  |
| S8 | “traits OR symptoms OR characteristics OR features”  (1980-2014; English) | **Search modes** - Boolean/Phrase | 1127983 |  |
| S6 | “prevalence OR occurrence OR frequency"(1980-2014; English) | **Search modes** - Boolean/Phrase | 789614 |  |
| S4 | “college students OR university students OR undergraduate OR postgraduate OR pupil"(1980-2014; English) | **Search modes** - Boolean/Phrase | 17966 |  |
| S2 | “borderline personality disorder OR BPD OR borderline personality"  (1980-2014; English) | **Search modes** - Boolean/Phrase | 4154 |  |

**None usable or new**

**CINAHL Plus**

| **Search**  **ID#** | **Search Terms** | **Search Options** | **Actions** | **Date** |
| --- | --- | --- | --- | --- |
| S7 | S5 AND S6 | **Search modes** - Boolean/Phrase | 2 | **30.7.15** |
| S6 | S3 AND S4 | **Search modes** - Boolean/Phrase | 30,179 |  |
| S5 | S1 AND S2 | **Search modes** - Boolean/Phrase | 40 |  |
| S4 | “traits OR symptoms OR characteristics OR features”  (1980-2014; English) | **Search modes** - Boolean/Phrase | 244,180 |  |
| S3 | “prevalence OR occurrence OR frequency"(1980-2014; English) | **Search modes** - Boolean/Phrase | 149,830 |  |
| S2 | “college students OR university students OR undergraduate OR postgraduate OR pupils"(1980-2014; English) | **Search modes** - Boolean/Phrase | 30,829 |  |
| S1 | “borderline personality disorder OR BPD OR borderline personality"  (1980-2014; English) | **Search modes** - Boolean/Phrase | 2,537 |  |

**None usable or new**

**Current contents connect (through Web of Science)**

| **Search**  **ID#** | **Search Terms** | **Search Options** | **Actions** | **Date** |
| --- | --- | --- | --- | --- |
| S3 | AND **TOPIC:** (prevalence OR occurrence OR frequency) | **Search modes** - Boolean/Phrase | 56 | **30.7.15** |
| S2 | **Refined by:** **TOPIC:** (college students OR university students OR undergraduates OR postgraduates or pupils) | **Search modes** - Boolean/Phrase | 260 |  |
| S1 | “**TOPIC:** (borderline personality disorder OR BPD OR borderline personality)  **Timespan:** 1998-2014. **Indexes:** ABES, SBS, CM, LS, PCES, ECT, AH, BC, EC. “ | **Search modes** - Boolean/Phrase | 8962 |  |

**None usable or new**

**EBM reviews** **(Allied and Complementary Medicine)**

| **No** | **Query Results** | **Results** | **Date** |
| --- | --- | --- | --- |
| #11 | #3 AND #6 | 3 | 29.7.15 |
| #10 | #3 AND #6 AND #9 | 2 |  |
| #9 | ''prevalence or occurrence or frequency” (date 1980-2014) limit 1 to english language [Limit not valid in CDSR,ACP Journal Club,DARE,CCTR,CLCMR; records were retained] | 55068 |  |
| #6 | “college students or university students or undergraduates or postgraduates or pupils” (date 1980-2014) limit 1 to english language [Limit not valid in CDSR,ACP Journal Club,DARE,CCTR,CLCMR; records were retained] | 2078 |  |
| #3 | “borderline personality disorder or BPD or borderline personality” (date 1980-2014)  limit 1 to english language [Limit not valid in CDSR,ACP Journal Club,DARE,CCTR,CLCMR; records were retained] | 704 |  |

**None usable or new**

**Embase**

| **No** | **Query Results** | **Results** | **Date** |
| --- | --- | --- | --- |
| #5 | #2 AND #3 AND #4 | 24 | 30.7.15 |
| #4 | prevalence OR occurrence OR frequency | **1,373,900** |  |
| #3 | college AND students OR university AND students OR undergraduates OR postgraduates OR pupils | **121,855** |  |
| #2 | Keyword search **"borderline personality disorder OR BPD or borderline personality"[MeSH Major Topic]** (date 1980-2014; English) | **8,269** |  |

**None usable or new**

**Google Scholar**

| **No** | **Query Results** | **Results** | **Date** |
| --- | --- | --- | --- |
| #2 | Date 1980-1980 | 0 | 30.7.15 |
| #1 | borderline personality disorder or borderline personality or bpd and college students or university students or undergraduates and postgraduates and prevalence | 210 |  |

**Ovid MEDLINE Search (PubMed)**

| **Search** | **Most Recent Queries** | **Date** | **Result** |
| --- | --- | --- | --- |
| #10 | Search **#4 AND #9** (date 1980-2014) | 30.7.15 | 11 |
| #9 | Search **#2 AND #6** (date 1980-2014) |  | 1031 |
| #8 | Search **traits OR symptoms OR characteristics OR features**(date 1980-2014) |  | 977438 |
| #6 | Search “**prevalence OR occurrence OR frequency”** (date 1980-2014; English) |  |  |
| #4 | Keyword search **"college students OR university students OR undergraduates OR postgraduates OR pupils"[MeSH Major Topic]** (date 1980-2014, English) |  | 29368 |
| #2 | Keyword search **"borderline personality disorder OR BPD or borderline personality"[MeSH Major Topic]** (date 1980-2014; English) |  | 8485 |

**None usable or new**

**Proquest Central**

| **Search** | **Most Recent Queries** | **Date** | **Result** |
| --- | --- | --- | --- |
| #3 | **(((borderline personality disorder) OR (bpd OR borderline personality)) AND (college students OR university students OR undergraduates OR postgraduates)) AND (prevalence OR occurrence)** (date 1980-2014; English) | 30.7.15 | 17 |
| #2 | ((borderline personality disorder) OR (bpd OR borderline personality)) AND (college students OR university students OR undergraduates OR postgraduates) |  | 255 |
| #1 | Keyword search **"borderline personality disorder OR BPD or borderline personality"[MeSH Major Topic]** (date 1980-2014; English) |  | 24,993 |

**None usable or new**

**PsycINFO Search**

| Search  ID# | **Search Terms** | **Actions** | **Date** |
| --- | --- | --- | --- |
| #11 | #9 AND #10 | 18 | 30.7.15 |
| #10 | #6 AND #9 | 18 |  |
| #9 | #2 AND #4 | 140 |  |
| #8 | “traits or symptoms or characteristics or features” | 482340 |  |
| #6 | “prevalence or occurrence or frequency” **(1980-2014; English)** | 198847 |  |
| #4 | “college students or university students or undergraduates or postgraduates or pupils” **(1980-2014; English)** | 139694 |  |
| #2 | “borderline personality disorder OR BPD or borderline personality" **(1980-2014; English)** | 8398 |  |

**None usable or new**

**PubMed**

| Search  ID# | **Search Terms** | **Actions** | **Date** |
| --- | --- | --- | --- |
| 4 | 1 AND 2 AND 3 **(1980-2014)** | 53 | 30.7.15 |
| 3 | (("epidemiology"[Subheading] OR "epidemiology"[All Fields] OR "prevalence"[All Fields] OR "prevalence"[MeSH Terms]) OR ("epidemiology"[Subheading] OR "epidemiology"[All Fields] OR "incidence"[All Fields] OR "incidence"[MeSH Terms])) OR ("epidemiology"[Subheading] OR "epidemiology"[All Fields] OR "occurrence"[All Fields] OR "epidemiology"[MeSH Terms] OR "occurrence"[All Fields]) AND ("1980/01/01"[PDAT] : "2014/12/31"[PDAT]) | **2646276** |  |
| 2 | (((college[All Fields] AND ("students"[MeSH Terms] OR "students"[All Fields])) OR (("universities"[MeSH Terms] OR "universities"[All Fields] OR "university"[All Fields]) AND ("students"[MeSH Terms] OR "students"[All Fields]))) OR undergraduate[All Fields]) AND postgraduate[All Fields] AND ("1980/01/01"[PDAT] : "2014/12/31"[PDAT]) | 139086 |  |
| 1 | (("borderline personality disorder"[MeSH Terms] OR ("borderline"[All Fields] AND "personality"[All Fields] AND "disorder"[All Fields]) OR "borderline personality disorder"[All Fields]) OR bpd[All Fields]) OR ("borderline personality disorder"[MeSH Terms] OR ("borderline"[All Fields] AND "personality"[All Fields] AND "disorder"[All Fields]) OR "borderline personality disorder"[All Fields] OR ("borderline"[All Fields] AND "personality"[All Fields]) OR "borderline personality"[All Fields]) AND ("1980/01/01"[PDAT] : "2014/12/31"[PDAT]) | 11635 |  |

**None usable or new**

**Scopus**

| Search  ID# | **Search Terms** | **Actions** | **Date** |
| --- | --- | --- | --- |
| 4 | 1 AND 2 AND 3 **>1979** | 32 | 30.7.15 |
| 3 | TITLE-ABS-KEY(prevalence OR occurrence OR frequency) AND PUBYEAR > 1993 | 3,067,731 |  |
| 2 | TITLE-ABS-KEY(university students OR college students OR undergraduates OR postgraduates) AND PUBYEAR > 1993 | 143,656 |  |
| 1 | TITLE-ABS-KEY (borderline personality disorder OR bpd OR borderline personality) (1980-2014) | 10175 |  |

**None usable or new**

**Taylor & Francis online**

| Search  ID# | **Search Terms** | **Actions** | **Date** |
| --- | --- | --- | --- |
| 4 | 1 AND 2 AND 3 (1980-2014) | 20 | 30.7.15 |
| 3 | Search Everything (prevalence OR occurrence OR frequency) AND PUBYEAR > 1993 | 766,155 |  |
| 2 | Search Everything (university students OR college students OR undergraduates OR postgraduates) AND PUBYEAR (1980-2014) | 1,338,024 |  |
| 1 | Search Everything (borderline personality disorder OR bpd OR borderline personality) (1980-2014) | 14854 |  |

**Web of Science**

| Search ID# | **Search Terms** | **Actions** | **Date** |
| --- | --- | --- | --- |
| S3 | Refined by: **TOPIC:** *(college students or university students or undergraduates or postgraduates or pupils)* AND **TOPIC:** *(prevalence OR occurrence OR frequency)*  Timespan=1980-2014. Indexes=SCI-EXPANDED, SSCI, A&HCI, CPCI-S, CPCI-SSH, BKCI-S, BKCI-SSH, CCR-EXPANDED, IC. | 47 | 30.7.15 |
| S2 | *(borderline personality disorder OR BPD or borderline personality)*  Refined by: **TOPIC:** *(college students or university students or undergraduates or postgraduates or pupils)*  Timespan=1980-2014. Indexes=SCI-EXPANDED, SSCI, A&HCI, CPCI-S, CPCI-SSH, BKCI-S, BKCI-SSH, CCR-EXPANDED, IC. | 294 |  |
| S1 | *(borderline personality disorder OR BPD or borderline personality)*  Timespan=1980-2014. Indexes=SCI-EXPANDED, SSCI, A&HCI, CPCI-S, CPCI-SSH, BKCI-S, BKCI-SSH, CCR-EXPANDED, IC. | 13,380 |  |

**None usable or new**

**Other databases searched:**

| **Database** | **Date Searched** | **Records per total search terms** |
| --- | --- | --- |
| AEI: Australian Education Index | 30.7.2015 Not available | 0 |
| AMI: Australasian medical index |  | 3 (0 relevant) |
| APAIS: Australian public affairs information service |  | 13 (0 relevant) |
| BMJ Best Practice (now Best Practice) |  | 0 |
| Cochrane Library |  | 7/8623 (0 relevant) |
| MIMS Online |  | 0 |
| Informit Online |  | 221 (0 relevant) |

Appendix 2B: Ten Step Literature Search

| 1. DATABASE SEARCHING  Multidisciplinary: Current Contents Connect, Embase, Google Scholar, Informit Online, Ovid MEDLINE, Proquest Central, Scopus, Taylor & Francis Online, Web of Science with conference procedings; Subject-specific: AMED, Biological Abstracts, CINAHL Plus, Cochrane Library, MIMS Online, PsycARTICLES, PsychINFO, PubMed;  n=880  (Journal Articles = 856; Book Sections = 3; Books = 21) |
| --- |
| 🡻 |
| 2. REMOVE DUPLICATES  Exclude n=43, retain n=813  (Journal Articles = 806; Book Sections = 1; Books = 6) |
| 🡻 |
| 3. SCREENING #1, BY TITLE  Exclude n=283a, retain n=523  (Journal Articles = 523) |
| 🡻 |
| 4. SCREENING #2, BY ABSTRACT  Exclude n=356b, retain n=167  (Journal Articles = 167) |
| 🡻 |
| 5. SCREENING #3, BY FULL TEXT  Exclude n=128c, retain n=39  (Journal Articles = 39) |
| 🡻 |
| 6. CITED REFERENCE SEARCHING ^d^  Science Citation Index Expanded (1994-2014) and Social Sciences Citation Index (1994-2014, via ISI Web of Science).  Add n=0, retain n=39  (Journal articles = 39) |
| 🡻 |
| 7. HAND SEARCHINGe  Journal of Personality Disorders and Psychopathology, 1994-2014  Add n=3, retain n=44  (Journal articles = 44) |
| 🡻 |
| 8. RECORDS ALREADY KNOWN, NOT FOUND IN OTHER SEARCHES  Add n=4, retain n=48  (Journal articles = 48f) |
| 🡻 |
| 9. CORRESPONDENCE WITH AUTHORSf  Exclude n=5, retain n=45 records (120 estimates)  (Journal articles = 43f) |
| 🡻 |
| 10. REPEAT OF ELECTRONIC SEARCHES PRIOR TO SUBMISSION FOR PUBLICATION  Add n=0, retain n=43 (50 estimates)  (Journal articles = 43) |

^a^ First Screening Exclusions: see appendix for details

^b^ Second Screening Exclusions: see appendix for details

^c^ Third Screening Exclusions - see appendix for details

^d^ No additional resources that were retained were located

^e^ Pavony et al, (2013) & Hocschild-Tolpin (2004).

^f^ After writing to authors, 5 journal articles were excluded after discovering they either reported on a sample existing in the review, or had methodological characteristics not reported in the record that met exclusion criteria.

Appendix 2C: Data Items and Explanations

| **Variable** | **Definition** |
| --- | --- |
| author | Author (first) of study |
| pubyear | Year record was published (1-1994-2000; 2=2001-2007; 3=2008-2014) |
| datayear | Year data was collected |
| gapyear | Number of years between data collection & publication |
| source | Source of record: (DB=database search; CRS=citation reference search; AK=already known)  articles; HS=hand search |
| country | Country data was collected within (USA; Canada; Poland; Spain; Taiwan; Turkey) |
| prevtotal | Reported % (as decimal) prevalence of clinically significant BPD |
| N | Total number of participants |
| studtype | Study level of students (UG = undergraduate; UG/PG = undergraduate & postgraduate; PG = postgraduate) |
| incentive | Incentive used (yes; no) |
| incentivetype | Type of incentive used (cash; course credit; none) |
| anon | Anonymity (anonymous; identifiable) |
| Research focus | Whether topic under study was BPD or other focus |
| responrat | Response rate % |
| toolname | Name of measure used to quantify BPD |
| periodsymp | Time period BPD symptoms were measured over (2 weeks, month, lifetime) |
| modemeasure | E.g. structured clinical interview or self report |
| No of items | Number of items in measure |
| respformat | Format of measure e.g. 3-point, 4-point, true/false, yes/no |
| trait/sym/features | Whether measure taps traits, symptoms or features |
| constructype | Whether items assessed BPD by presence of item, frequency of item, or veracity (e.g. true, very true) of item |
| clincut | clinical cutoffs of measure |
| authcut | Clinical cutoffs used by authors relative to measures |
| cutchange | whether cutoffs had been changed by authors (yes; no) |
| cutchangno | Numerical difference in cut off change (numerical continuous) |
| agerangelow | Minimum age of participants |
| agerangehigh | Maximum age of participants |
| meanage | Mean age of participants |
| SDage | Standard deviation of mean age of participants |
| Female | Proportion of female participants % |
| Male | Proportion of male participants % |
| Fem/male | Gender (female =0, male =1) |
| white | Proportion of white participants % |
| black | Proportion of African/ black participants % |
| hispan | Proportion of Hispanic/Latino participants % |
| asian | Proportion of Asian participants % |
| other | Proportion of 'other' ethnic participants % |

Appendix 2D: Study Characteristics

| **Citation** | **Country** | **Ince-**  **ntive** | **Sample** | **N** | **M Age**  **(SD)** | **Measure** | **Period** | **Anon-**  **imity** | **Collection**  **Format** | **Q-Format**  **/type** | **Cut-**  **off** | **Prev** | **95%**  **LCI** | **95%**  **UCI** |
| --- | --- | --- | --- | --- | --- | --- | --- | --- | --- | --- | --- | --- | --- | --- |
| Abramson, et al., (1998) | USA | No | UG | 342 | 19.8  (3.0) | IPDE | Life | Identifi-  able | Structured interview | 3-point  frequency | 10 | 2.3 | 1.2 | 4.6 |
| Alemany-Martinez, et al., (2008) | Spain | No | PG | 78 | 30  (NR) | IPDE | Life | Anony-mous | SR | 3-point  frequency | 10 | 32.1 | 22.7 | 43.1 |
| Ayduk, et al, (2008) | USA | Yes | UG | 379 | 21.2  (3.6) | PAI-BOR | Life | Identifi-able | SR | 4-point  veracity | 38 | 14.5 | 11.3 | 18.4 |
| Bagge et al., (2004) | USA | Yes | UG | 351 | 20  (0) | PAI-BOR | Life | Identifi-able | SR | 4-point  veracity | 38 | 1.7 | 0.8 | 3.8 |
| Bracken-Minor & Devitt-Murphy  (2014) | USA | Yes | UG | 480 | 21.3  (5.7) | MSI-BPD | Life | Anony-mous | SR | yes/no  veracity | 7 | 14.2 | 11.3 | 17.6 |
| Cheavens et al., (2012) | USA | Yes | UG | 330 | 19.6  (2.3) | PAI-BOR | Life | Potent- ially | SR | 4-point  veracity | 38 | 17.3 | 13.6 | 21.7 |
| Chen et al., (2011) | USA | Yes | UG | 197 | 21.8  (6.2) | PDI-IV | Life | Identifi-able | Structured interview | 3-point  frequency | 10 | 13.2 | 9.1 | 18.7 |
| Chien et al., (2011) | Taiwan | No | UG | 2731 | 19.2  (2.4) | ASRI-4 | Life | Identifi-able | SR | 4-point  severity | 6 | 0.5 | 0.3 | 0.9 |
| Cierpiałkowska & Pasikowski (2013) | Poland | No | UG | 134 | NR | BPI-T20 | Life | Anony-mous | SR | true/false  veracity | 20 | 27.6 | 20.7 | 35.8 |
| Geiger, et al (2014) | USA | Yes | UG | 181 | 18.9  (1.1) | PAI-BOR | Life | Identif- iable | SR | 4-point  veracity | 37 | 18.8 | 13.7 | 25.1 |
| **Citation** | **Country** | **Ince-**  **ntive** | **Sample** | **N** | **M Age**  **(SD)** | **Measure** | **Period** | **Anon-**  **imity** | **Collection**  **Format** | **Q-Format**  **/type** | **Cut-**  **off** | **Prev** | **95%**  **LCI** | **95%**  **UCI** |
| Glenn & Klonsky (2009) | USA | Yes | UG | 273 | NR | MSI-BPD | Life | Identif- iable | SR | yes/no  veracity | 7 | 12.3 | 8.8 | 17.1 |
| Gratz, Breetz & Tull (2009) | USA | Yes | UG | 392 | 20.3  (2.46) | BEST | 1 Month | Anony-mous | SR | 5-point  severity | 30 | 25.5 | 21.4 | 30.1 |
| Helfritz & Sanford (2006) | USA | Yes | UG | 41 | 20.0  (1.4) | PAI-BOR | Life | Identif- iable | SR | 4-point  veracity | 38 | 4.9 | 1.2 | 17.5 |
| Herr et al., (2013) | USA | Yes | UG | 98 | 18.7  (1.2) | PAI-BOR | Life | Anony-mous | SR | 4-point  veracity | 7 | 24.5 | 17.0 | 33.9 |
| Hochschild Tolpin (2004) | USA | Yes | UG | 296 | 19.4  (1.8) | PAI-BOR | Life | Anony-mous | SR | 4-point  veracity | 10 | 10.1 | 7.2 | 14.1 |
| Hong et al., (2011) | USA | Yes | UG | 234 | 18.6  (1.2) | PAI-BOR | Life | Identif- iable | SR | 4-point  veracity | 38 | 25.2 | 20.1 | 31.2 |
| Klonsky (2008) | USA | Yes | UG/PG | 45 | 20.1  (1.4) | MSI-BPD | Life | Identif- iable | SR | yes/no  veracity | 7 | 9.9 | 6.8 | 14.0 |
| Krupnick et al., (2004) | USA | Yes | UG | 209 | 20.4  (NR) | SCID-II | Life | Identif- iable | Structured Interview | yes/no  present | 10 | 1.9 | 0.7 | 5.0 |
| Lewis, et al., (2001) | USA | Yes | UG | 240 | 19.0  (1.8) | PAI-BOR | Life | Anony-mous | SR | 4-point  veracity | 38 | 13.8 | 9.9 | 18.7 |
| MacLaren & Best (2010) | USA | Yes | UG | 153 | 24.8  (8.1) | NEO-PI-R | Life | Anony-mous | SR | 5-point  veracity | 4 | 7.3 | 4.1 | 12.7 |
| Pavony & Lenzenweger (2013) | USA | Yes | UG/  PG | 667 | 19.3  (2.5) | IDPE-S | Life | Anony-mous | SR | 3-point  frequency | 38 | 4.9 | 1.6 | 14.2 |
| **Citation** | **Country** | **Ince-**  **ntive** | **Sample** | **N** | **M Age**  **(SD)** | **Measure** | **Period** | **Anon-**  **imity** | **Collection**  **Format** | **Q-Format**  **/type** | **Cut**  **-off** | **Prev** | **95%**  **LCI** | **95%**  **UCI** |
| Peters et al., (2013) | USA | Yes | UG | 227 | 19.4  (3.0) | PAI-BOR | Life | Anony-mous | SR | 4-point  veracity | 37 | 11.0 | 9.2 | 13.2 |
| Presniak, et al., (2010) | Canada | Yes | UG | 674 | 20.0  (2.5) | PAI-BOR | Life | Anony-mous | SR | 4-point  veracity | 38 | 7.9 | 5.7 | 10.9 |
| Reich, et al., (2013) | USA | Yes | UG | 818 | 19.1  (3.3) | PAI-BOR | Life | Anony-mous | SR | 4-point  veracity | 42 | 4.1 | 2.9 | 5.8 |
| Ruiz, et al. (1999) | USA | Yes | UG | 355 | 19.0  (2.3) | PDQ-R | Life | Anony-mous | SR | true/false  veracity | 6 | 10.1 | 7.4 | 13.7 |
| Ryan & Sheehan (2007) | USA | Yes | UG | 1418 | 18.2  (NR) | PDQ-4 | Life | Anony-mous | SR | true/false  veracity | 38 | 12.5 | 10.9 | 14.3 |
| Sansone, et al., (1994) | USA | Yes | UG | 33 | 20.6  (1.7) | PDQ-R | Life | Anony-mous | SR | true/false  veracity | 5 | 27.0 | 14.8 | 44.7 |
| Sar et al.,  (2006) | Turkey | No | UG | 1301 | 19.1  (NR) | SCID-II | Life | Identif- iable | Structured Interview | yes/no  presence | 5 | 8.5 | 7.1 | 10.2 |
| Sauer & Baer (2010) | USA | Yes | UG | 519 | 18.0  (0) | PAI-BOR | Life | Identif- iable | SR | 4-point  veracity | 65 | 17.1 | 14.1 | 20.6 |
| Stepp et al., (2005) | USA | Yes | UG | 5000 | 20.8  (4.2) | PAI-BOR | Life | Identif- iable | SR | 4-point  veracity | 38 | 3.9 | 3.4 | 4.5 |
| Taylor,  (2005) | USA | Yes | UG | 123 | 19.0  (1.5) | SIDP-IV | Life | Identifi-  able | Structured Interview | yes/no  presence | 10 | 4.1 | 1.7 | 9.4 |
| Taylor et al. (2008) | USA | Yes | UG | 2085 | 19.0  (3.9) | SCID-II-Q | Life | Identifi-  able | SR | yes/no  presence | 5 | 7.0 | 6.0 | 8.2 |
| **Citation** | **Country** | **Ince-**  **ntive** | **Sample** | **N** | **M Age**  **(SD)** | **Measure** | **Period** | **Anon-**  **imity** | **Collection**  **Format** | **Q-Format**  **/type** | **Cut-**  **off** | **Prev** | **95% LCI** | **95%**  **UCI** |
| Thompson et al., (2012) | USA | Yes | UG | 180 | NR | PAI-BOR | Life | Identifi-  able | SR | 4-point  veracity | 70 | 3.3 | 1.5 | 7.2 |
| Tragesser & Benfield (2012) | USA | Yes | UG | 225 | 20.4  (5.2) | PAI-BOR | Life | Anony-mous | SR | 4-point  veracity | 37 | 13.3 | 9.5 | 18.4 |
| Tragesser et al. (2013) | USA | Yes | UG | 606 | 19.3  (1.8) | PAI-BOR | Life | Anony-  mous | SR | 4-point  veracity | 37 | 1.2 | 0.6 | 2.4 |
| Trull (1995) | USA | Yes | UG | 1697 | 19.0  (1.4) | PAI-BOR | Life | Anony-mous | SR | 4-point  veracity | 37 | 14.2 | 12.6 | 15.9 |
| Valentiner et al., (2014) | USA | Yes | UG | 329 | 19.2  (1.8) | MSI-BPD | Life | Anony-mous | SR | yes/no  veracity | 7 | 10.6 | 7.7 | 14.5 |
| Watson &  Sinha (1998) | USA | No | UG | 1729 | 20.4  (4.4) | CATI | Life | Anony-mous | SR | true/false  veracity | 48 | 4.0 | 3.2 | 5.0 |
| Werner & Crick (1999) | USA | Yes | UG | 225 | 19.5  (NR) | PAI-BOR | Life | Anony-mous | SR | 4-point  veracity | 37 | 4.9 | 2.7 | 8.6 |
| Wright, et al., (2010) | USA | Yes | UG | 258 | 18.9  (0.9) | IPDE-S | Life | Identifi-  able | SR | 3-point  frequency | 10 | 1.6 | 0.6 | 4.1 |
| Wupperman et al., (2008) | USA | Yes | UG | 342 | 19.0  (5.1) | PAI-BOR | Life | Anony-mous | SR | 4-point  veracity | 38 | 10.8 | 7.9 | 14.6 |
| Yalch et al., (2012) | USA | Yes | UG | 235 | NR | PAI-BOR | Life | Anony-mous | SR | 4-point  veracity | 4 | 29.8 | 24.3 | 35.9 |
| Zeigler-Hill & Abraham (2006) | USA | Yes | UG | 123 | 19.0  (2.1) | PAI-BOR | Life | Anony-mous | SR | 4-point  veracity | 38 | 14.1 | 9.5 | 20.5 |

### ASRI-4= Adult Self-report Inventory-4; BEST=Borderline Evaluation of Severity Over Time; BPI-T20 = Borderline Personality Inventory; BSL-23=Borderline Symptom List -23 item; CATI = Coolidge Axis II Inventory; Frequency = frequency of item; IDPE= International Personality Disorder Examination Structured Interview; IPDE-S = IPDE Screening Questionnaire; LCI = Lower Confidence Interval; MSI-BPD = McLean Screening Instrument for Borderline Personality Disorder; NEO-PI-R= NEO Personality Inventory Revised; NR= Not reported; PAI-BOR= Personality Assessment Inventory–Borderline Features Scale; PDE = Personality Disorders Examination; PDI-IV = Personality Disorder Interview for DSM-IV; PDQ-4= Personality Diagnostic Questionnaire 4th-Edition; PDQ-R= Personality Diagnostic Questionnaire – Revised; Presence = whether item present or not; Prev = Prevalence; SCID-II = Structured Clinical Interview for DSM-III-R / DSM-IV personality disorders; SIDP-IV= Structured Interview for DSM-IV Personality; UCI = Upper Confidence Interval; USA = United States of America; Veracity = whether item is “true” or not

Appendix 2E: Full Citations of Studies Included in the Review

1. Abramson, L. Y., Alloy, L. B., Hogan, M. E., Whitehouse, W. G., Cornette, M., Akhavan, S., & Chiara, A. (1998). Suicidality and cognitive vulnerability to depression among college students: a prospective study. *Journal of Adolescence, 21*(4), 473-487. doi: http://dx.doi.org/10.1006/jado.
2. Alemany Martínez, A., Berini Aytés, L., & Gay Escoda, C. (2008). The burnout syndrome and associated personality disturbances. The study in three graduate programs in Dentistry at the University of Barcelona. *Medicina Oral, Patología Oral y Cirugia Bucal, 2008, vol. 13, num. 7, p. 444-450*. doi: none
3. Ayduk, Ö., Zayas, V., Downey, G., Cole, A. B., Shoda, Y., & Mischel, W. (2008). Rejection sensitivity and executive control: Joint predictors of borderline personality features. *Journal of Research in Personality*, *42*(1), 151-168. doi: http://dx.doi.org.ezproxy.lib.monash.edu.au/10.1016/j.jrp.2007.04.002
4. Bagge, C., Nickell, A., Stepp, S., Durrett, C., Jackson, K., & Trull, T. J. (2004). Borderline personality disorder features predict negative outcomes 2 years later. *Journal of Abnormal Psychology*, *113*(2), 279. doi: 10.1037/0021-843X.113.2.279
5. Bracken-Minor, K. L., & McDevitt-Murphy, M. E. (2013). Differences in features of non-suicidal self-injury according to borderline personality disorder screening status. *Archives of suicide research*, (accepted). doi: 10.1080/13811118.2013.809040
6. Cheavens, J. S., Strunk, D. R., & Chriki, L. (2012). A Comparison of Three Theoretically Important Constructs: What Accounts For Symptoms of Borderline Personality Disorder? *Journal of Clinical Psychology, 68*(4), 477-486. doi: 10.1002/jclp.20870.
7. Chen, Y., Huprich, S. K., & Hsiao, W. (2011). Affect regulation and depressive personality disorder. Journal of Personality Disorders, 25(6), 755-64. doi:http://dx.doi.org/101521pedi2011256755
8. Chien, Y. L., Gau, S. S. F., & Gadow, K. D. (2011). Sex difference in the rates and co-occurring conditions of psychiatric symptoms in incoming college students in Taiwan. *Comprehensive psychiatry*, *52*(2), 195-207. doi:10.1016/j.comppsych.2010.03.009
9. Cierpiałkowska, L., Marszał, M., & Pieniążek, M. (2012). Defensive functioning in individuals with borderline personality organization in the light of empirical re-search. *Pol J Appl Psychol*, *10*(1), 7-19. No doi
10. Geiger, P. J., Peters, J. R., & Baer, R. A. (2014). Using a measure of cognitive distortion to examine the relationship between thought suppression and borderline personality features: A multi-method investigation. *Personality and Individual Differences*, *59*, 54-59. doi: http://dx.doi.org.ezproxy.lib.monash.edu.au/10.1016/j.paid.2013.11.005
11. Glenn, C. R., M.A., & Klonsky, E. D. (2009). Emotion dysregulation as a core feature of borderline personality disorder. *Journal of Personality Disorders, 23*(1), 20-8. Retrieved from http://search.proquest.com/docview/195241794?accountid=12528
12. Gratz, K. L., Breetz, A., & Tull, M. T. (2010). The moderating role of borderline personality in the relationships between deliberate self‐harm and emotion‐related factors. *Personality and Mental Health*, *4*(2), 96-107. doi: 10.1002/pmh.102
13. Helfritz, L. E., & Stanford, M. S. (2006). Personality and psychopathology in an impulsive aggressive college sample. *Aggressive Behavior*, *32*(1), 28-37. doi: 10.1002/ab.20103
14. Herr, N. R., Keenan-Miller, D., Rosenthal, M. Z., & Feldblum, J. (2013). Negative interpersonal events mediate the relation between borderline features and aggressive behavior: Findings from a nonclinical sample of undergraduate women. *Personality Disorders: Theory, Research, and Treatment*, *4*(3), 254-260. doi: 10.1037/a0032212
15. Hong, P. Y., Ilardi, S. S., & Lishner, D. A. (2011). The aftermath of trauma: The impact of perceived and anticipated invalidation of childhood sexual abuse on borderline symptomatology. *Psychological Trauma: Theory, Research, Practice, and Policy*, *3*(4), 360-368. doi: 10.1037/a0021261
16. Hochschild Tolpin, L., Cimbolic Gunthert, K., Cohen, L. H., & O'neill, S. C. (2004). Borderline Personality Features and Instability of Daily Negative Affect and Self‐Esteem. *Journal of Personality*, *72*(1), 111-138. doi: 10.1111/j.0022-3506.2004.00258.x
17. Klonsky, E. D. (2008). What is emptiness? Clarifying the 7th criterion for borderline personality disorder. *Journal of personality disorders*, *22*(4), 418-426. doi: 10.1521/pedi.2008.22.4.418
18. Krupnick, J. L., Green, B. L., Stockton, P., Goodman, L., & al, e. (2004). Mental health effects of adolescent trauma exposure in a female college sample: Exploring differential outcomes based on experiences of unique trauma types and dimensions. *Psychiatry, 67*(3), 264-79. Retrieved from http://search.proquest.com/docview/220668805?accountid=12528
19. Lewis, S. F., Fremouw, W. J., Del Ben, K., & Farr, C. (2001). An investigation of the psychological characteristics of stalkers: Empathy, problem-solving, attachment and borderline personality features. *Journal of Forensic Sciences*, *46*(1), 80-84. doi: none
20. MacLaren, V. V., & Best, L. A. (2010). Nonsuicidal self-injury, potentially addictive behaviors, and the Five Factor Model in undergraduates. *Personality and Individual Differences*, *49*(5), 521-525. doi: http://dx.doi.org/10.1016/j.paid.2010.05.019
21. Pavony, M. T., & Lenzenweger, M. F. (2013). Somatosensory processing and borderline personality disorder features: a signal detection analysis of proprioception and exteroceptive sensitivity. *Journal of Personality Disorders*, *27*(2), 208-221. doi: 10.1521/pedi_2013_27_076
22. Peters, J. R., Geiger, P. J., Smart, L. M., & Baer, R. A. (2013). Shame and Borderline Personality Features: The Potential Mediating Role of Anger and Anger Rumination. *Personality Disorders: Theory, Research, and Treatment*,*5*(1), 1-9.DOI: 10.1037/per0000022
23. Presniak, M. D., Olson, T. R., & MacGregor, M. W. (2010). The role of defense mechanisms in borderline and antisocial personalities. *Journal of personality assessment*, *92*(2), 137-145. doi: 10.1080/00223890903510373
24. Reich, D. B., Zanarini, M. C., Hopwood, C. J., Thomas, K. M., & Fitzmaurice, G. M. (2013). Comparison of affective instability in borderline personality disorder and bipolar disorder using a self-report measure. *Personality and Mental Health*, *8,* 143-150. doi: 10.1002/pmh.1247
25. Ruiz, M. A., Pincus, A. L., & Bedics, J. B. (1999). Using the structural analysis of social behavior (SASB) to differentiate young adults with borderline personality disorder features. *Journal of Personality Disorders, 13*(2), 187-198. doi:http://dx.doi.org/10.1521/pedi.1999.13.2.187
26. Ryan, K., & Shean, G. (2007). Patterns of interpersonal behaviors and borderline personality characteristics. *Personality and Individual Differences*, *42*(2), 193-200. doi:10.1016/j.paid.2006.06.010
27. Sansone, R. A., Fine, M. A., & Nunn, J. L. (1994). A Comparison of Borderline Personality Symptomatology and Self-Destructive Behavior in Women With Eating, Substance Abuse, and Both Eating and Substance Abuse Disorders. *Journal of Personality Disorders, 8*(3), 219-228. doi: http://dx.doi.org/10.1521/pedi.1994.8.3.219
28. Sar, V., Akyuz, G., Kugu, N., Ozturk, E., & Ertem-Vehid, H. (2006). Axis I dissociative disorder comorbidity in borderline personality disorder and reports of childhood trauma. *Journal of Clinical Psychiatry*, *67*(10), 1583-1590. doi: 10.4088/JCP.v67n1014
29. Sauer, S. E., & Baer, R. A. (2010). Validation of measures of biosocial precursors to borderline personality disorder: Childhood emotional vulnerability and environmental invalidation. *Assessment*, *17*(4), 454-466. doi: 10.1177/1073191110373226
30. Stepp, S. D., Trull, T. J., & Sher, K. J. (2005). Borderline personality features predict alcohol use problems. *Journal of personality disorders*, *19*(6), 711-722. doi: 10.1521/pedi.2005.19.6.711
31. Taylor, J. (2005). Substance use disorders and cluster B personality disorders: Physiological, cognitive, and environmental correlates in a college sample. *The American Journal of Drug and Alcohol Abuse*, *31*(3), 515-535. doi: 10.1081/ADA-200068107
32. Taylor, J., James, L. M., Bobadilla, L., & Reeves, M. D. (2008). Screening for disinhibited disorder cases in a college population: Performance of the SMAST, DAST, SCID-II-Q, and PDQ-4. *Psychological Assessment, 20*(4), 351-360. doi: 10.1037/a0013452
33. Thompson, R. J., Payne, S. C., Horner, M. T., & Morey, L. C. (2012). Why borderline personality features adversely affect job performance: The role of task strategies. *Personality and Individual Differences, 52*(1), 32-36. doi: http://dx.doi.org/10.1016/j.paid.2011.08.026
34. Tragesser, S. L., & Benfield, J. (2012). Borderline personality disorder features and mate retention tactics. *Journal of Personality Disorders*, *26*(3), 334-344.doi: 10.1521/pedi.2012.26.3.334
35. Tragesser, S. L., Jones, R. E., Robinson, R. J., Stutler, A., & Stewart, A. (2013). Borderline Personality Disorder Features and Risk for Prescription Opioid Use Disorders. *Journal of personality disorders*, *27*(4), 427-441. doi: 10.1521/pedi_2013_27_094
36. Trull, T. J. (1995). Borderline personality disorder features in nonclinical young adults: 1. Identification and validation. *Psychological Assessment*, *7*(1), 33-41. doi: 10.1037/1040-3590.7.1.33
37. Valentiner, D. P., Hiraoka, R., & Skowronski, J. J. (2014). Borderline personality disorder features, self-verification, and committed relationships. *Journal of Social and Clinical Psychology, 33*(5), 463-480. doi: http://dx.doi.org/101521jscp2014335463
38. Watson, D. C., & Sinha, B. K. (1998). Comorbidity of DSM‐IV personality disorders in a nonclinical sample. *Journal of clinical psychology*, *54*(6), 773-780. doi: 10.1002/(SICI)1097-4679(199810)54:6<773::AID-JCLP3>3.0.CO;2-I
39. Werner, N. E., & Crick, N. R. (1999). Relational aggression and social-psychological adjustment in a college sample. *Journal of Abnormal Psychology*, *108*(4), 615-23. doi:10.1037/0021-843X.108.4.615
40. Wright, A. G., Pincus, A. L., & Lenzenweger, M. F. (2010). Modeling stability and change in borderline personality disorder symptoms using the Revised Interpersonal Adjective Scales–Big Five (IASR–B5). *Journal of Personality Assessment*, *92*(6), 501-513. doi: 10.1080/00223891.2010.513288
41. Wupperman, P., Neumann, C. S., & Axelrod, S. R. (2008). Do deficits in mindfulness underlie borderline personality features and core difficulties?. *Journal of Personality Disorders*, *22*(5), 466-482. doi: 10.1521/pedi.2008.22.5.466
42. Yalch, M. M., Thomas, K. M., & Hopwood, C. J. (2012). The veracity of trait, symptom and prototype approaches for describing borderline and antisocial personality disorders. *Personality and Mental Health*, *6*(3), 207-216. doi: 10.1002/pmh.1184
43. Zeigler–Hill, V., & Abraham, J. (2006). Borderline personality features: instability of self–esteem and affect. *Journal of Social and Clinical Psychology*, *25*(6), 668-687. doi: 10.1521/jscp.2006.25.6.668

Appendix 2F: List of Studies Reporting on the Same Sample

* indicates the paper included in the review, others not included

***Glenn, C. R., M.A., & Klonsky, E. D. (2009). Emotion dysregulation as a core feature of borderline personality disorder. *Journal of Personality Disorders, 23*(1), 20-8. Retrieved from http://search.proquest.com/docview/195241794?accountid=12528**

Glenn, C. R., & Klonsky, E. D. (2010). A multimethod analysis of impulsivity in nonsuicidal self-injury. *Personality Disorders: Theory, Research, and Treatment*, *1*(1), 466-473. doi: 10.1002/jclp.20661

Glenn, C. R., Weinberg, A., & Klonsky, E. D. (2009). Relationship of the Borderline Symptom List to DSM-IV Borderline Personality Disorder Criteria Assessed by Semi-Structured Interview. *Psychopathology*, *42*(6), 394-398. doi: 10.1159/000241195

***Peters, J. R., Geiger, P. J., Smart, L. M., & Baer, R. A. (2013). Shame and Borderline Personality Features: The Potential Mediating Role of Anger and Anger Rumination. *Personality Disorders: Theory, Research, and Treatment*,*5*(1), 1-9.DOI: 10.1037/per0000022**

Peters, J. R., Upton, B. T., & Baer, R. A. (2013). Brief Report: Relationships Between Facets of Impulsivity and Borderline Personality Features. *Journal of Personality Disorders*, *27*(4), 547-552. doi: 10.1521/pedi_2012_26_044

Peters, J. R., Eisenlohr-Moul, T. A., Upton, B. T., & Baer, R. A. (2013). Nonjudgment as a moderator of the relationship between present-centered awareness and borderline features: Synergistic interactions in mindfulness assessment. *Personality and Individual Differences*, *55*(1), 24-28. doi: http://dx.doi.org/10.1016/j.paid.2013.01.021

***Sar, V., Akyuz, G., Kugu, N., Ozturk, E., & Ertem-Vehid, H. (2006). Axis I dissociative disorder comorbidity in borderline personality disorder and reports of childhood trauma. *Journal of Clinical Psychiatry*, *67*(10), 1583-1590. doi: 10.4088/JCP.v67n1014**

Sar, V., Alioğlu, F., Akyuz, G., & Karabulut, S. (2014). Dissociative Amnesia in Dissociative Disorders and Borderline Personality Disorder: Self-Rating Assessment in a College Population. *Journal of Trauma & Dissociation*, *15*(4), 477-93. doi: 10.1080/15299732.2014.902415

***Sauer, S. E., & Baer, R. A. (2010). Validation of measures of biosocial precursors to borderline personality disorder: Childhood emotional vulnerability and environmental invalidation. *Assessment*, *17*(4), 454-466. doi: 10.1177/1073191110373226**

Sauer-Zavala, S. E., Geiger, P. J., & Baer, R. A. (2013). The Effect of Anger Rumination in the Relationship Between Borderline Personality Disorder Symptoms and Precursors. *Journal of Personality Disorders*, *27*(4), 465-472. doi: 10.1521/pedi_2013_27_098

*** Tragesser, S. L., & Benfield, J. (2012). Borderline personality disorder features and mate retention tactics. *Journal of Personality Disorders*, *26*(3), 334-344.doi: 10.1521/pedi.2012.26.3.334**

Tragesser, S. L., & Robinson, R. J. (2009). The role of affective instability and UPPS impulsivity in borderline personality disorder features. *Journal of Personality Disorders*, *23*(4), 370-383. doi: 10.1521/pedi.2009.23.4.370

***Trull, T. J. (1995). Borderline personality disorder features in nonclinical young adults: 1. Identification and validation. *Psychological Assessment*, *7*(1), 33-41. doi: 10.1037/1040-3590.7.1.33**

Trull, T. J., Useda, D., Conforti, K., & Doan, B. T. (1997). Borderline personality disorder features in nonclinical young adults: 2. Two-year outcome. *Journal of Abnormal Psychology*, *106*(2), 307-314. doi: 10.1037/0021-843X.106.2.30

Appendix 2G: Screening Exclusions General Exclusion Criteria

- 1. prevalence % of clinically significant Borderline Personality Disorder not reported or unable to be calculated e.g. n reported in clinical range as a % of N;
  2. not university or college samples;
  3. language other than English;
  4. samples from prisons, school or clinical settings;
  5. adolescents or school-aged children;
  6. psychiatric inpatients or outpatients;
  7. clinical practice guidelines or recommendations;
  8. genetic, molecular, or cellular level studies;
  9. editorials, reviews, qualitative studies, case-control or case studies;
  10. clinical trials or evaluations of interventions, management strategies or treatments.
  11. unpublished dissertations or theses

Screening #1 Exclusions

1. prevalence of BP symptoms, features or traits in clinically significant range not reported (n=172 excluded);
2. not in English (n=6 excluded);
3. clinical populations (n=45 excluded);
4. adolescent/school populations (n=62 excluded);
5. prison populations (n=23 excluded);
6. community samples (n=18 excluded);
7. subjects (total 148 excluded)
8. case-studies (n=4 excluded)
9. case-control in design (n=10 excluded)
10. clinical trials or evaluations of interventions, management strategies or treatments (n=91 excluded);
11. duplicates (n=22 excluded);

*Notes: exclusions sum to greater than 694 because as a number of articles were excluded on the basis of more than one criterion.*

Screening #2 Exclusions

1. prevalence of BP symptoms, features or traits in clinically significant range not reported (n=274 excluded);
2. not in English (n=6 excluded);
3. clinical populations (n=32 excluded);
4. adolescent/school populations (n=47 excluded);
5. prison populations (n=42 excluded);
6. community samples (n=32 excluded);
7. subjects (total 159 excluded)
8. case-studies (n=16 excluded)
9. case-control in design (n=10 excluded)

*Notes: exclusions sum to greater than 356 because some articles were excluded on the basis of more than one criterion.*

Screening #3 Exclusions

1. prevalence of BP symptoms, features or traits in clinically significant range not reported (n=132 excluded);
2. clinical populations (n=x excluded);
3. adolescent/school populations (n=x excluded);
4. prison populations (n=18 excluded);
5. community samples (n=21 excluded);
6. subjects (total 326 excluded)
7. duplicates (n=11 excluded);

Appendix 2H: Data Extraction Form

| **Data Extraction Form** | | | | | | | | | | | | | |
| --- | --- | --- | --- | --- | --- | --- | --- | --- | --- | --- | --- | --- | --- |
|  | **Complete?** | | **Included? (reason if not)** | | | | | | | | |  |  |
| **Systematic Review** | Yes |  | Yes | |  |  |  |  |  |  | |  |  |
| **Meta-analysis** | Yes |  | Yes | |  |  |  |  |  |  | |  |  |
| Source | | | | | | | | | | | | | |
| Report ID | J01 |  |  |  |  |  |  |  |  |  |  | |  |
| Study ID | S01 |  |  |  |  |  |  |  |  |  |  | |  |
| Article type (i.e. journal  article) | Journal article | |  |  |  |  |  |  |  |  |  | |  |
| Citation (author, pub year, title, journal) | Abramson et al., 1998. Suicidality and cognitive vulnerability to depression among college students: a prospective study | | | | | | | | | | | | |
| Context | | | | | | | | | | | | | |
| Purpose (purpose of current study/ objective / study  problem) | Using a behavioral high-risk two-site prospective design, we tested the cognitive vulnerability hypotheses about suicidality | | | | | | | | | | | | |
| Context (context in which px answered questions/ in  which data was collected/ project name, i.e. was it a health and lifestyle study) | Freshmen less than 30 years old participated in two phase questionnaire | | | | | | | | | | | | |
| Country (state/city) | USA (North-eastern) | | |  |  |  |  |  |  |  | |  |  |
| Data collection year | 1992 |  |  |  |  |  |  |  |  |  | |  |  |
| Methods | | | | | | | | | | | | | |
| Design | Longitudinal | |  |  |  |  |  |  |  |  | |  |  |
| Sampling/ recruitment | A random sample of 5378 freshman from 2 unis were screened using paper q’naires. Obtained through classes dormitories, campus activities, and campus advertisements. Participants were excluded from the final sample if they met criteria for any mood or anxiety disorder, psychosis, and bipolar. | | | | | | | | | | | | |
| Incentive/reward | None | |  |  |  |  |  |  |  |  | |  |  |
| Response rate | 30% |  |  |  |  |  |  |  |  |  | |  |  |
| Participants | | | | | | | | | | | | | |
| Sample type | Undergraduates | | | | | | | |  |  | |  |  |
| Sample size |  | All |  |  |  |  |  | F |  |  | |  | M |
|  | 170 | |  |  |  | N= 116 | | |  | 68.2 | | N=54 | 31.8 |
| Ethnicity (%) | White/ Cauc. | | Hisp/  Latino | |  |  | Asian | |  | Black/  African | |  | Other |
|  | 80 |  | 2.1 |  | 3.8 | |  |  |  | 14.1 | |  | 0 |
| SES (income) | NR |  |  |  |  |  |  |  |  |  | |  |  |
| SES (education) | At least secondary school | | | |  |  |  |  |  |  | |  |  |
| SES (occupation) | Students | |  |  |  |  |  |  |  |  | |  |  |
| Mental health history | Answered questions about recent psychological distress, suicidal-related  behaviours and help-seeking behaviours | | | | | | | | | | | | |
| Age |  | M |  |  |  | SD | |  |  | MIN | |  | MAX |
|  | 22 |  |  | 3.0 | |  |  |  |  | 18 | |  | 22 |

| Measure |  | | | | | |
| --- | --- | --- | --- | --- | --- | --- |
| Construct measured | DSM-IV-TR BPD traits | | | | | |
| Definition/ instruction told to px/ wording of  item | Frequency of symptom – 3-point | | | | | |
| Q’re Name | IPDE | | | | | |
| Q’re Type (paper,  internet, interview, SAQ) | Structured Clinical interview | | | | | |
| Identifiable | Participant completely identifiable | | | | | |
| Time period/s | Life | | | | | |
| Clinical cut-off | 5 – as per DSM diagnostic cut-off | | | | | |
| Clinical cutoff changed? | No | | | | | |
| Number of items/or item deletion? | 9/No | | | | | |
| Results |  | | | | | |
|  | All | | F | | M | |
| Time period | N | % | N | % | N | % |
| Life | 8/342 | 2.4 |  |  |  |  |

Appendix 2I: BPD in University Populations: Publications over Time

Appendix 2J: Tools Used to Measure BPD among Studies in Review

| Name of Questionnaire | N | % |
| --- | --- | --- |
| Personality Assessment Inventory–Borderline Features Scale (PAI-BOR; Morey, 1991) | 22 | 48.9 |
| McLean Screening Instrument for Borderline Personality Disorder (MSI-BPD; Zanarini et al., 2003) | 5 | 11.1 |
| The International Personality Disorder Examination (IPDE; Loranger et al., 1994) | 4 | 8.9 |
| Structured Clinical Interview (SCID-II; First et al., 1997) | 3 | 6.7 |
| Personality Diagnostic Questionnaire – Revised (PDQ-R; Hyler et al., 1990) | 2 | 4.4 |
| Personality Diagnostic Questionnaire- 4^th^ edition (PDQ-4; Hyler, 1994) | 1 | 2.2 |
| Adult Self-report Inventory-4 (ASRI-4; Gadow et al, 2008) | 1 | 2.2 |
| Borderline Evaluation of Severity Over Time (BEST; Pfohl, 2009) | 1 | 2.2 |
| Borderline Symptom List -23 item (BSL-23; Bohus, 2009) | 1 | 2.2 |
| Coolidge Axis II Inventory (CATI; Coolidge, 1992) | 1 | 2.2 |
| NEO Personality Inventory Revised (NEO-PI-R; Costa & McCrae, 2010) | 1 | 2.2 |
| Personality Disorder Interview for DSM-IV (PDI-IV; Widiger et al., 1995) | 1 | 2.2 |
| Borderline Personality Inventory (BPI; Leichsenring, 1999) | 1 | 2.2 |
| Structured Interview for DSM-IV Personality (SIDP-IV; Pfohl et al, 1997) | 1 | 2.2 |
